# The accuracy of emergency weight estimation systems in children—a systematic review and meta-analysis

**DOI:** 10.1186/s12245-017-0156-5

**Published:** 2017-09-21

**Authors:** Mike Wells, Lara Nicole Goldstein, Alison Bentley

**Affiliations:** 1 Division of Emergency Medicine, Faculty of Health Sciences, University of the Witwatersrand, 7 York Road, Parktown, Johannesburg, 2193 South Africa; 2 Postnet Suite 429, Private Bag X1510, Glenvista, 2058 South Africa

**Keywords:** Weight estimation, Broselow tape, PAWPER tape, Mercy method

## Abstract

The safe and effective administration of fluids and medications during the management of medical emergencies in children depends on an appropriately determined dose, based on body weight. Weight can often not be measured in these circumstances and a convenient, quick and accurate method of weight estimation is required. Most methods in current use are not accurate enough, but the newer length-based, habitus-modified (two-dimensional) systems have shown significantly higher accuracy. This meta-analysis evaluated the accuracy of weight estimation systems in children. Articles were screened for inclusion into two study arms: to determine an appropriate accuracy target for weight estimation systems; and to evaluate the accuracy of existing systems using standard meta-analysis techniques. There was no evidence found to support any specific goal of accuracy. Based on the findings of this study, a proposed minimum accuracy of 70% of estimations within 10% of actual weight (PW10 > 70%), and 95% within 20% of actual weight (PW20 > 95%) should be demonstrated by a weight estimation system before being considered to be accurate. In the meta-analysis, the two-dimensional systems performed best. The Mercy method (PW10 70.9%, PW20 95.3%), the PAWPER tape (PW10 78.0%, PW20 96.6%) and parental estimates (PW10 69.8%, PW20 87.1%) were the most accurate systems investigated, with the Broselow tape (PW10 55.6%, PW20 81.2%) achieving a lesser accuracy. Age-based estimates achieved a very low accuracy. Age- and length-based systems had a substantial difference in over- and underestimation of weight in high-income and low- and middle-income populations. A benchmark for minimum accuracy is recommended for weight estimation studies and a PW10 > 70% with PW20 > 95% is suggested. The Mercy method, the PAWPER tape and parental estimates were the most accurate weight estimation systems followed by length-based and age-based systems. The use of age-based formulas should be abandoned because of their poor accuracy.

**Electronic supplementary material:**

The online version of this article (10.1186/s12245-017-0156-5) contains supplementary material, which is available to authorized users.

## Introduction

It cannot be considered to be good medical practice to use a weight estimation system that is known to be inaccurate [1]. When children’s weight cannot be measured during emergency care, an accurate, rapid estimation of weight is needed, as the safety and effectiveness of emergent interventions may ultimately depend on the accuracy of the weight estimation [2, 3]. Since most drug doses in children are based on weight, an accurate estimation of weight is important to ensure that a correct amount of medication is administered to achieve the desired effect, as well as to prevent the potential complications and side-effects of overdosing [4, 5]. This is relevant because most paediatric medication errors occur in the Emergency Department and most cases of resultant patient harm are related to incorrect dosing [6–8].

The problem is that most contemporary methods used to estimate children’s weight have been shown to lack sufficient accuracy and consistency of performance in different populations [9]. Most existing weight estimation systems are “one-dimensional”, because a single variable, usually age or length, is used in the weight estimation methodology. These systems fail because a single variable cannot adequately account for the biological variability of weight-for-age and weight-for-length [10, 11]. There is a wide variability of body habitus that is not accounted for in these weight-estimation systems, aggravated by the increasing levels of obesity affecting children [12, 13]. Newer, more promising, methods are the “two-dimensional” or dual length- and habitus-based systems, which include two variables in the estimation methodology: length (or a surrogate such as humerus or ulna length) and habitus (or a surrogate such as mid-arm circumference or waist circumference) [5, 14–17]. These have been shown to be much more accurate than the older, one-dimensional systems, in many studies [5, 15, 18–22].

Healthcare providers may also need more than one approach to emergency weight estimation: while parental estimates of weight can be very accurate, parents may not be present at the time that emergency care is required (especially in the prehospital environment) [9]. In these situations, an evidence-based alternative system may be required.

There has been a large amount of material published on weight estimation in children. It would be useful to combine the data from these studies to establish the accuracy of different methodologies both within and between different populations. Since many of the same weight estimation systems are used in populations with very different prevalences of underweight and obese children, it needs to be ascertained whether this impacts on the accuracy outcomes of these systems.

In order to create an evidence-based approach to emergency paediatric weight estimation, it is crucial to discover which methods predict weight most accurately and which are most appropriate for emergency use. This will enable clinicians to decide which systems they should incorporate into their clinical practice and will provide some guidance to those who administer, teach and train paediatric advanced life support on which systems are important.

The overall aim of this study was to determine which paediatric weight estimation systems most accurately estimate total body weight in children. The first objective was to determine whether there was evidence in the literature for an acceptable benchmark level of accuracy for a weight estimation system. The second objective was to extract and pool data on the performance of paediatric weight estimation systems to integrate the findings, provide a more comprehensive analysis on their functioning and identify those systems that operated best in diverse populations. The third objective was to directly compare the accuracy of paediatric weight estimation systems, for which paired data was available, using pooled data and meta-analysis techniques.

Only one meta-analysis has addressed this topic, but was limited to studies in low- and middle-income countries [23].

## Methods

This systematic review and meta-analysis followed the PRISMA guidelines.

## Search strategy

Online databases (MEDLINE, SCOPUS, Science Direct and Google) were interrogated for eligible studies, published between January 1983 and May 2017, using the following search terms: “paediatric weight estimation”, “weight estimation children” and “Broselow tape”. Citation lists of reviewed papers were examined for additional relevant articles. Studies in any language were included if English translations were obtainable. To minimise publication bias, all studies with adequate reporting were included, whether full-text articles, dissertations, abstracts, conference presentations or other unpublished data that had undergone some form of peer-review.

## Study selection and eligibility criteria

**Fig. 1:**
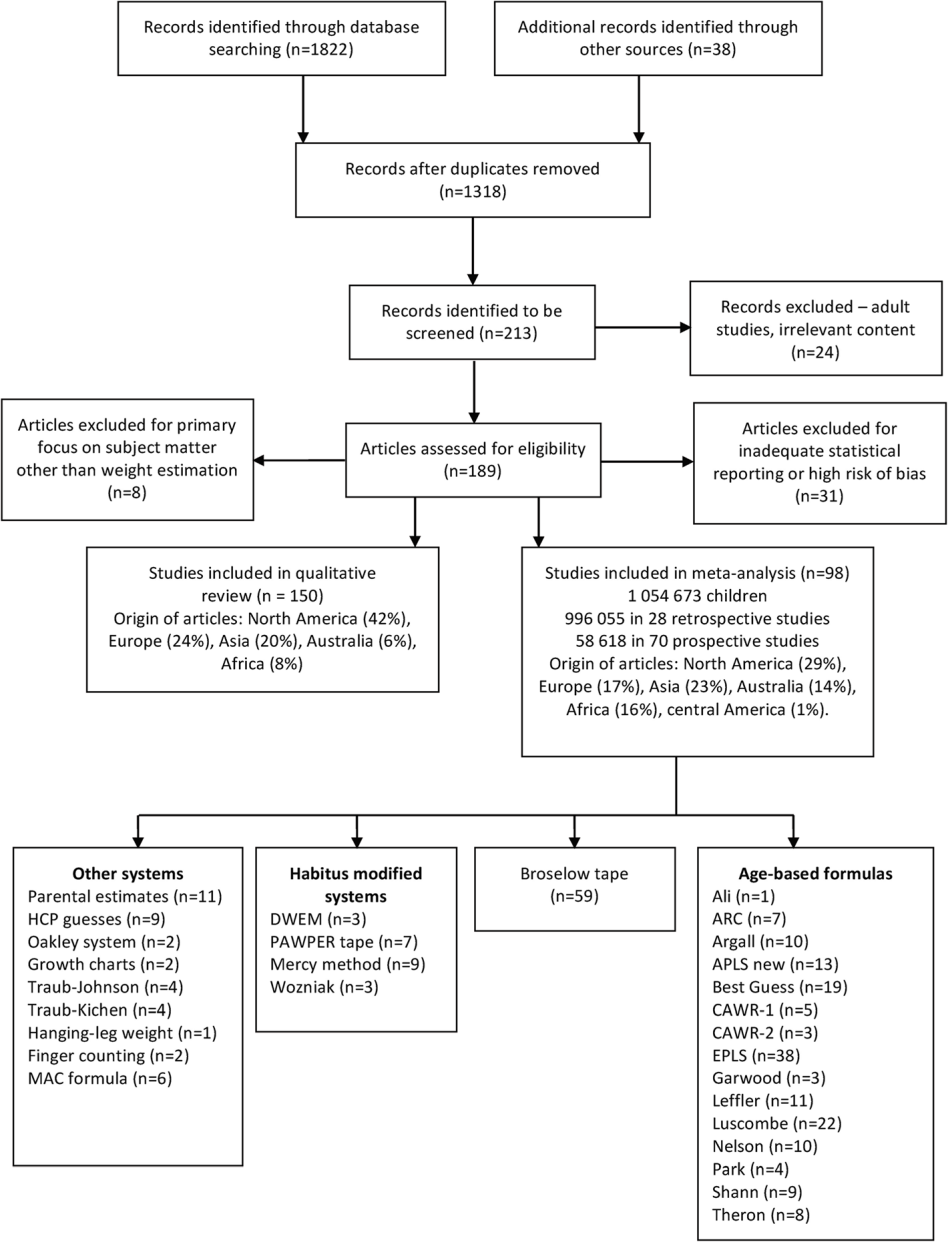
The PRISMA flow-chart of the study design

All studies that evaluated weight-estimation methodologies were assessed for inclusion into the study by two separate investigators (MW and LG). Articles that contained discussions on desired targets of accuracy of weight estimation systems, or analysis of the performance of weight-estimation systems were included in the qualitative arm of the review. Studies that presented original data with either accuracy data (percentage of estimations within 10% of actual weight (PW10)) or bias and precision data (mean percentage error plus an appropriate indicator of variance), or both, were included in the meta-analysis. Studies that did not include original data, those that did not include usable data and those at high risk of bias (see below) were excluded from the meta-analysis (see Fig. 1).

## Data abstraction and analysis

Data was extracted from the included studies independently by two researchers (MW, LG), cross-checked and confirmed. Standard statistics for meta-analysis of method-comparison studies were used [24], with an emphasis on evaluating accuracy (percentage of estimations within 10% of actual weight), bias (mean percentage error) as well as precision (limits of agreement of percentage error). Two methods of representing the pooled parametric and non-parametric data were employed: a fixed effects model weighted by inverse variance and a random effects model. In general, the random effects model was preferred because of the large variance within and between samples as well as the effects of several very large database studies that may have introduced bias.

Many of the evaluated studies presented incomplete data. Where it was possible, without risking bias, missing data was imputed using standard methodologies [25].

Direct comparisons between weight estimation systems, using pooled paired data, were performed with non-parametric techniques based on PW10 accuracy data, where such data was available.

## Subgroup analysis

There was considerable heterogeneity in the use and composition of subgroups within the included studies. Wherever possible, subgroup analyses that had been performed in each study were included in the overall meta-analysis. The included subgroups focused on different age groups as previous studies have shown a difference in weight estimation accuracy between infants (<1 year), toddlers and pre-school children (1 to 6 years) and older children (>6 years of age) [26].

## Risk of bias within and across studies

Reporting bias was minimised by including all available methodologically sound studies (published or not). Methodological causes of potential bias were common (e.g. the Broselow tape was not actually used in many studies, but weight-estimates were generated from length data), but these were individually assessed and rated according to the level of risk of systematic bias. Studies with a high risk of bias were excluded from the meta-analysis (e.g. studies which excluded children above or below certain weight-for-length centiles).

## Sensitivity analysis

There were three large database studies among those evaluated, with more than 100,000 children, one of which had more than 400,000 data points [27–29]. The effects of these “virtual” weight estimation studies, from very large databases, were carefully considered to establish any significant contribution to bias or distorted outcomes.

## Software

Statistical analysis was performed using Stata (StataCorp. 2015. Stata Statistical Software: Release 14. College Station, TX: StataCorp LP), Graphpad Prism (GraphPad Prism version 8.00 for Mac, GraphPad Software, La Jolla, California, USA, www.graphpad.com) and Review manager (Review Manager (RevMan) [Computer program]. Version 5.3. Copenhagen: The Nordic Cochrane Centre, The Cochrane Collaboration, 2014).

## Results

### Excluded studies

The most common reason for exclusion of potentially relevant studies was incomplete data presentation (see Fig. 1). The large database studies did not have a significant impact on overall outcomes based on the sensitivity analysis and were therefore not excluded from the analysis.

### Characteristics of included studies

Two-thirds of included studies evaluated multiple weight-estimation systems and contained paired data or made direct comparisons, while one-third evaluated only a single system. Prospective studies accounted for the majority of articles (70/98 (71.4%)) but a minority of total patients (58,618/1,054,673 (5.6%)).

Table 1 provides a descriptive summary of the studies included in both the qualitative review as well as the meta-analysis, including the major findings and limitations of each study and the risk of bias assessment for each included study.

**Table 1:** Studies included in the qualitative review and quantitative meta-analysis

Author and date	Study size (*N*)	Country	Design	Patient ages	Estimation techniques evaluated	Target	Arm	Risk of bias	Major findings; comments; major limitations
Traub 1983 [69]	> 20,000	USA	R	0 to 18 years	Formula to estimate IBW (Traub-Kichen formula)	None	1	Low	Findings: Height was a good predictor of weight; IBW is only useful for a handful of drugs; TBW must be used in low weight-for-height children. Comments: Derivation study for Traub-Kichen formula. IBW predicted (actually 50th centile weight-for-length) by formula. Limitations: Incomplete presentation of data. Limited validation of formula.
Garland 1986 [67]	258	USA	P	0 to 19 years	DWEM, weight table	< 10%*	1, 2	Low	Findings: DWEM performed best of methods tested. Body habitus accurately assessed by evaluators. Comments: First ever report of evaluation of weight estimation systems in the literature. None of the systems tested were very accurate. Limitations: Only children up to 170 cm were included. Incomplete presentation of data.
Lubitz 1988 [70]	937	USA	P	0 to 12 years	Broselow tape	None	1, 2	Low	Findings: Broselow tape better than healthcare provider guesses and similar accuracy to DWEM. Accuracy of Broselow tape falls off sharply in children > 25 kg. Comments: Original study of Broselow tape. Authors recommended that an assessment of body habitus in children > 25 kg should be considered. Limitations: No formal, prospective comparison with other methodologies or indication of desired accuracy.
Oakley 1988 [71]	–	UK	–	–	–	None	1	N/A	Findings: Reference chart needed to aid rapid and accurate management. Comments: Weight estimation table derived from averaged boy-girl 50th centile weight-for-height (source not mentioned). Limitations: No validation of methodology.
Losek 1989 [72]	–	–	–	–	–	None	1	N/A	Findings: “Body habitus + height = accurate weight estimate”. Comments: Letter claiming superior performance of the DWEM over the Broselow tape. No original data. Limitations: No mention of desired accuracy.
Haftel 1990 [73]	100	USA	P	2 months to 15 years	Hanging-leg weight	None	1, 2	Low	Findings: System accurate in children > 10 kg and more so > 25 kg. Comments: Good results never evaluated in subsequent studies. Limitations: Small sample size. Incomplete presentation of data.
Hughes 1990 [74]	139	UK	P	0 to 10 years	Broselow tape, healthcare provider guesses	None	1	N/A	Findings: Broselow tape performed substantially better than nurses’ guesses. Comments: First validation study of Broselow tape in the UK. Limitations: Small sample size. Incomplete presentation of data.
Greig 1997 [36]	75	UK	P	0 to 12 years	healthcare provider guesses	None	1, 2	Low	Findings: Guesses of weight are very inaccurate; children should be weighed whenever possible. Comments: Authors suggest that accurate weight estimation is required for most drugs administered in emergency situations. Age-based formulas were wrongly considered acceptable. Limitations: Incomplete presentation of data, very small sample.
Leffler 1997 [75]	117	USA	P	0 to 5 years	Parental estimates, Leffler	< 10%^†^	1, 2	Low	Findings: Parental estimates performed much better than formula. Distraught parents may be unreliable. Comments: Small sample size. Only children < 6 years included. Limitations: Over- or underestimation not recorded. Incomplete presentation of data.
Dearlove 1999 [76]	50	UK	P	1 to 16 years	Parental estimates, Broselow tape, EPLS, Argall	< 10%^†^	1, 2	Low	Findings: Broselow tape performed best, far better than parental estimates and age-based formulas. Comments: The target of 10% accuracy chosen for children was deliberately less than the 20% that the authors considered would be appropriate for adults. Limitations: Incomplete presentation of data and small sample size.
Goldman 1999 [77]	233	Israel	P	–	Parental estimates	< 10%^†^	1, 2	Low	Findings: Parents, especially mothers, can accurately estimate their children’s weights. Comments: Those parents that had weighed their children an average of 5 weeks previously had the best results. The authors defined highly accurate weight estimations as < 5% error, accurate as < 10% error and semi-accurate as < 20% error. Limitations: Incomplete presentation of data. Misinterpretation of bias as indicative of accuracy.
Harris 1999 [78]	100	USA	P	0 to 8 years	Parental estimates, healthcare provider guesses	None	1, 2	Low	Findings: Weight estimates by parents, nurses and doctors were significantly unreliable. The error is “so great and so frequent that clinically significant untoward effects can be anticipated”. Comments: Broselow tape recommended by authors. Limitations: Incomplete presentation of data.
Molyneux 1999 [53]	142	Malawi	P	8 months to 5 years	Blantyre tape, healthcare provider guesses	<20%^†^	1, 2	Low	Findings: Healthcare provider guesses were very inaccurate; Blantyre tape better than guesses. A 20% error considered an acceptable target. Comments: Very young study population, mostly under 5 years. Limitations: Incomplete presentation of data.
Kun 2000 [79]	909	Hong Kong	P	0 to 12 years	Broselow tape	<10%*	1, 2	Low	Findings: Broselow tape most accurate in children from 10 to 25 kg, but acceptable for all children. Adjustment for habitus would be advantageous. Comments: Accuracy of Broselow tape outside of the 10–25 kg range was actually poor. The accuracy in this range was reasonable, but not as good as the authors suggest. Limitations: Poor interpretation of statistics. Broselow tape version not reported.
Carroll 2001 [80]	169	UK	P	–	EPLS, novel methods	None	1	N/A	Findings: MAC and shoe size were better indicators of weight than age. Comments: Abstract. Interesting concept, frequently cited abstract. Limitations: Incomplete presentation of data.
Vilke 2001 [81]	80	USA	P	–	Broselow tape, healthcare provider guesses	<50%	1	N/A	Findings: 95% of estimates within acceptable error range. Comments: Unrealistic target range, with no evidence basis. Tenfold errors in drug doses in 10% of cases; Broselow tape more accurate than guesses. Limitations: Incomplete presentation of data.
Black 2002 [52]	495	Australia	P	0 to 18 years	EPLS, Broselow tape, DWEM, Oakley, TJ, TK	None	1, 2	Low	Findings: Broselow tape and DWEM were more accurate than formulas. These methods should be used if weighing not possible. Comments: EPLS worst performer but poor accuracy of all systems. Good reproducibility of assessment of body habitus. Limitations: Incomplete presentation of data. Broselow tape version not reported.
Hofer 2002 [82]	585	Switzerland	R	6 months to 11 years	Broselow tape	<10%*	1, 2	Low	Findings: Broselow tape was accurate but underestimated weight in older children. Comments: Nearly 25% of sample excluded because they were too tall for the tape. Limitations: Broselow tape not actually used and version not reported. Incomplete presentation of data.
Uesegi 2002 [83]	48	Japan	P	–	Healthcare provider guesses	<20%	1, 2	Low	Findings: Doctors’ guesses of children’s weight were not accurate—drug doses should therefore be titrated in small paediatric patients. Comments: Wide variation in different doctors’ accuracy, not related to seniority. All estimators were very inaccurate; worst estimations occurred in children < 20 kg. Limitations: Incomplete presentation of data; conclusion that underestimation of weight may “not be a serious problem” was not supported by the evidence.
Argall 2003 [84]	300	UK	P	1 to 10 years	EPLS, Broselow tape	None	1	N/A	Findings: Both methods performed poorly and worsened with increasing age. Comments: Difficult to draw any conclusions from this study, but Broselow tape marginally better than formula. Authors suggest that methods of weight estimation not keeping up with increasing obesity. Limitations: Broselow tape version not reported. Incomplete presentation of data.
Potier 2003 [85]	–	–	–	–	EPLS, Broselow tape	None	1	N/A	Findings: EPLS “may” be losing accuracy with increasing obesity; Broselow tape “may indeed be” more accurate. Comments: Mini-PICO analysis. Limitations: Limited qualitative-only evaluation. No comment on acceptable degree of accuracy.
Hohenhaus 2004 [86]	–	–	–	–	Broselow tape	None	1	N/A	Findings: Broselow tape may cause significant weight estimation errors if used incorrectly. Broselow tape more intended for equipment size determination than for weight estimation. Limitations: Broselow tape proposed as best instrument with minimal discussion. No targets for weight estimation.
Moore 2004 [87]	144	USA	P	–	Broselow tape	None	1	N/A	Findings: Despite using Broselow tape, only 56% correct medication doses delivered. Comments: Despite improving weight estimation, the Broselow tape did not decrease medication errors. Limitations: Incomplete presentation of data.
Luscombe 2005 [88]	237	UK and New Zealand	P	1 to 10 years	EPLS, Leffler	None	1, 2	Low	Findings: The Leffler formula was more accurate than the EPLS formula. Comments: A new formula was promoted but the evidence was limited: only bias was evaluated, not accuracy. Both formulas underestimated weight significantly The Luscombe formula, as known today, was the result of a later study. Limitations: Incomplete presentation of data.
Theron 2005 [89]	909	New Zealand	P	1 to 10 years	EPLS, Shann, Leffler, Oakley, Broselow tape	None	1, 2	Low	Findings: Formulas and Broselow tape underestimated the weight of Pacific Island and Maori children. Comments: Broselow tape was the best performer of the systems tested, despite the fact that the authors reported the contrary. Limitations: Broselow tape version not reported. Incomplete presentation of data.
Bavdekar 2006 [90]	500	India	P	0 to 2 years	Novel formula based on foot length	None	1	N/A	Findings: Foot length can be used for emergency drug calculation Comments: Only 10% of population > 1 year old. very poor statistics. Method of data analysis made findings unreliable and uninterpretable - bias confused with accuracy.
Nieman 2006 [37]	7813	USA	R	0 to 12 years	Broselow tape	< 10%*	1, 2	Low	Findings: One third of children had inaccurate weight estimations. The 1998 tape performed better than the 2002 tape. A measurement of obesity (e.g. MAC) should be added to Broselow tape to increase accuracy. Comments: Broselow tape version 1998 and 2002A used. The study population BMI was 16.8. Good statistics. Limitations: Broselow tape not actually used. Nearly 7% of sample excluded because too tall for the tape.
Varghese 2006 [91]	500	India	P	1 to 12 years	Argall, EPLS, Nelson, Broselow tape	None	1	N/A	Findings: Formulas overestimated weight in this developing-world study. The Broselow tape was the most accurate. Comments: More than half the study population was under 6 months of age and only 8% > 5 years. Limitations: Incomplete presentation of data.
Anderson 2007 [92]	–	–	–	–	–	None	1	N/A	Comments: Letter. The author comments on benefits of under- vs. overestimation of weight. No quantification of permissible or safe error was suggested.
Dieckman 2007 [93]	–	–	–	–	–	None	1	N/A	Comments: Editorial on weight estimation and drug dosing. LBW might be better than TBW. No evidence offered for opinion; no weight estimation target suggested (for TBW or LBW).
Du Bois 2007 [94]	400	USA	P	0 to 19 years	Broselow tape, DWEM	MPE < 5%	1, 2	Low	Findings: DWEM better than Broselow tape, but both systems underestimated weight especially in children > 20 kg. Comments: Good inter-rater reliability for habitus assessment. Performance of DWEM much better than Broselow tape, but both systems performed poorly. Limitations: Incomplete presentation of data. Broselow tape version not reported. Statistically inappropriate accuracy target.
Hashikawa 2007 [32]	1207	USA	R	0 to 12 years	Broselow tape	< 20%^†^	1	N/A	Findings: Approximately 60% accuracy of colour zones assignment (accurate drug dosing). Weight underestimated in obese and older children. Comments: Rising prevalence of obesity blamed for poor performance. Broselow tape version 2002B. The average study population BMI was 17. Limitations: Broselow tape not actually used. Assessment of correct zone assignment only, weight not measured. Incomplete presentation of data.
Im 2007 [95]	454	Korea	P	–	Broselow tape	None	1	High	Findings: Broselow tape accurate in children of normal weight-for-length Comments: Only children falling within 3rd to 97th weight-for-height centiles included. Very young study population. Broselow tape only recommended by authors for “normal-growth” children < 20 kg and < 120 cm. Limitations: Incomplete presentation of data. Conclusions not supported by findings.
Jang 2007 [96]	665	Korea	R	–	Broselow tape	< 10%	1, 2	Low	Findings: Broselow tape reasonably accurate in this population, but less so in children > 25 kg. Comments: Overall underestimation of weight. Performance not very good and on par with most other studies. Limitations: Broselow tape not actually used and version not reported. Incomplete presentation of data.
Kelly 2007 [97]	410	Australia	P	1 to 11 years	BG	< 20%	1, 2	Low	Findings: BG performed moderately well, but overestimated weight in low BMI children. Comments: Multiple papers on same data. BMI was 17 in study population. Significant number of children had large errors of weight estimation. Limitations: Incomplete presentation of data.
Krieser 2007 [98]	410	Australia	P	1 to 10 years	Parental estimates, Broselow tape, BG, Argall, EPLS	< 10%*	1, 2	Low	Findings: Parental estimates performed best, followed by Broselow tape. Only 11% of parents could not provide an estimate. Formulas performed much worse than other methods. Comments: Multiple papers on same data. Study population BMI was 17.1. Limitations: Broselow tape not actually used. Broselow tape version not reported. Incomplete presentation of data.
Luscombe 2007 [1]	13,988	UK	R	1 to 10 years	EPLS, Luscombe	None	1, 2	Low	Findings: The authors commented that since few children with high-acuity conditions are actually weighed in clinical practice, weight estimation essential. The EPLS formula significantly underestimated weight, which may lead to under-resuscitation. The Luscombe formula was more accurate. Comments: Both formulas actually performed poorly. Limitations: Incomplete presentation of data. Mean bias used incorrectly.
Luten 2007 [99]	–	–	–	–	Broselow tape	None	1	N/A	Findings: No substantiation for setting acceptable accuracy of weight estimation at 10%. Comments: Editorial comment; no evidence provided.
Nguyen 2007 [100]	410	Australia	P	1 to 11 years	Argall	< 10%*	1, 2	Low	Findings: Argall formula performed poorly, especially in children > 35 kg. Comments: Study population BMI was 17. Limitations: Incomplete presentation of data.
Patel 2007 [101]	360	USA	P	1 month to 10 years	Parental estimates, EPLS, Broselow tape	None	1	N/A	Findings: Parental estimates performed best, then Broselow tape, then EPLS. Comments: Abstract. Conclusions based on correlation and rudimentary statistical analysis. Limitations: Incomplete presentation of data.
Pollock 2007 [48]	100	Malawi	P	1 to 7 years	EPLS, Luscombe	None	1, 2	Low	Findings: The Luscombe formula was less accurate than EPLS with greater overestimation of weight. The authors suggested length-based systems should rather be used. Comments: Scientific letter. Both formulas performed very poorly with significant overestimation of weight. Limitations: Incomplete presentation of data.
Thompson 2007 [34]	1843	Australia	R	0 to 14 years	BG, EPLS, ARC	None	1, 2	Low	Findings: The BG formula performed better than EPLS and ARC formulas. Authors advised cautious use in infants. Comments: Data obtained from high acuity patients. The BG did not actually perform that well in this study. Limitations: Incomplete presentation of data. Only measures of bias used for comparison.
Tinning 2007 [102]	67,363	Australia	R	0 to 14 years	BG development study	None	1	N/A	Findings: New formulas developed with no target accuracy and no validation sample. Comments: Limited indication of accuracy of formulas as only measures of bias reported. Limitations: Incomplete presentation of data.
Zikos 2007 [103]									Comments: Identical abstract and data to Patel 2007
Gardner 2008 [104]	not reported	UK	P	1 to 15 years	EPLS, Luscombe, healthcare provider guesses	None	1	N/A	Findings: Age-based formulas often used as basis for weight guess, but the formulas were better than guesses. Comments: Abstract. Limitations: Incomplete presentation of data.
Luten 2008 [3]	–	–	–	–	–	None	1	N/A	Comments: This was a commentary on Broselow tape—that IBW might be better than TBW as a goal; “the Broselow tape is a tool that was not designed to be used without clinical judgement”. No evidence offered to support opinion; no goal target suggested (for TBW or IBW).
Ramarajan 2008 [56]	548	India	P	0 to 12 years	Broselow tape	< 10%^†^	1, 2	Low	Findings: Broselow tape overestimated weight by > 10% in Indian children over 10 kg. A correction factor was developed, but not validated. Comments: One of the few studies to show overestimation of weight by Broselow tape. Limitations: Broselow tape version not reported. Incomplete presentation of data.
Wells 2008 [105]	–	–	–	–	–	None	1	N/A	Comments: Letter. The Broselow tape has not been shown to accurately predict IBW. TBW should be used for dose calculation in emergencies.
Zink 2008 [106]	127	USA	P	0 to 17 years	Parental estimates, healthcare provider guesses, DWEM, Broselow tape	None	1	High	Findings: Broselow tape and DWEM were the least accurate methods. Comments: No conclusions can be drawn from this study because of the methodology. Limitations: The data appears to favour healthcare provider and parent guesses, but the statistical methodology is flawed.
Anstett 2009 [107]	545	Ireland	R	–	Broselow tape	None	1, 2	Low	Findings: The Broselow tape was often inaccurate and tended to underestimate weight. Comments: Abstract. The Broselow tape actually performed better in this study than in many other studies. Limitations: Broselow tape version not reported. Broselow tape not actually used.
Cattamanchi 2009 [108]	15,000	India	P	2 months to 12 years	Broselow tape	< 10%*	1, 2	Low	Findings: The Broselow tape performed well, especially in children < 10 kg but underestimated all others, especially in children > 18 kg. Comments: Abstract. Very large prospective study. The authors recommended a new version of Broselow tape for Indian children because of underestimation of weight. Limitations: Broselow tape version not reported. Incomplete presentation of data.
Cattermole 2009 [109]	1368	Hong Kong	P	1 to 12 years	MAC, Broselow tape, foot length	None	1	N/A	Findings: Estimates of weight can be based on MAC. A special colour-coded MAC tape could be produced to aid drug dosing. The authors recommended habitus modified use of Broselow tape. Comments: Abstract. No data presented. Broselow tape performed better in younger children, MAC better in older children. Limitations: No data presentation.
Partridge 2009 [110]	777	USA	P	0 to 20 years	Parental estimates, healthcare provider guesses	< 10%^†^	1, 2	Low	Findings: Parents were better than nurses at estimating weight; nurses were very inaccurate. Comments: Guessed weights most often underestimations. The longer the time from last weighing, the greater the error. All nurses, regardless of training and experience, were poor estimators. Limitations: Incomplete presentation of data.
Paw 2009 [111]	791	UK	P	1 to 12 years	EPLS	None	1	High	Findings: Very poor performance of EPLS formula. The authors recommended the Broselow tape or an alternative formula. Comments: Abstract. Uniformly abysmal accuracy across different ethnic groups. Limitations: Incomplete presentation of data.
Sandell 2009 [112]	846	UK	P	1 to 11 years	EPLS, age-based estimates vs. length-based estimates	None	1, 2	Low	Findings: Length-based and age-based systems are suitable in emergencies, but length-based were better; new formulas more accurate than EPLS; “one size fits all” approach not likely to be successful. Comments: Unique method of analysing data—does not allow comparisons with other studies in this format. Age-based methods less accurate than suggested; biological variability less in length-based than age-based systems. Limitations: Incomplete presentation of data.
So 2009 [113]	1011	USA	P	0 to 10 years	Broselow tape, Leffler, Theron	None	1, 2	Low	Findings: Broselow tape most accurate weight estimation method with best accuracy in normal BMI children. Comments: Study population BMI was 17.8. New formula developed, but not tested. Limitations: Incomplete presentation of data. Bias mistaken for accuracy. Broselow tape not actually used and Broselow tape version not reported.
Stewart 2009 [30]	475	Australia	P	0 to 10 years	Broselow tape	< 20%^†^	1, 2	Low	Findings: Best performance of Broselow tape between 10 and 25 kg but very inaccurate in children >25 kg. More accurate than age-based formulas, however. Comments: Masters dissertation. Good statistics. Limitations: Broselow tape version not reported.
Yamamoto 2009 [12]	542	Hawaii	P	–	Broselow tape, novel habitus-specific equation	None	1	High	Findings: Adding body habitus data to length increases accuracy of weight estimation. Comments: Useful data unable to be imputed from poorly presented statistics; overweight children were preferentially selected in this study Limitations: Incomplete presentation of data. Broselow tape version not reported and Broselow tape not actually used.
Bicer 2010 [114]	2319	Turkey	P	3 to 18 years	EPLS, Argall, novel formulas	None	1	High	Findings: Argall formula performed best. Comments: Article in Turkish; many formulas studied, often applied outside of the age-range for which they were intended. Limitations: Incomplete presentation of data. Measures of bias mistaken for measures of accuracy.
Casey 2010 [115]	1426	Australia	P	0 to 14 years	EPLS, BG, Broselow tape	< 20%^†^	1, 2	Low	Findings: BG was accurate in children 1–4 years of age. The EPLS formula was the least accurate of all methods. The authors recommend that the accuracy and ease-of-use of the Broselow tape mandates its use in the ED and pre-hospital. Comments: The authors’ suggestions that the BG was more “accurate” than the Broselow tape are misleading: the Broselow tape actually significantly outperformed the BG formula. BMI was 17.9 in study population. Limitations: Incomplete presentation of data. Broselow tape not actually used and Broselow tape version not reported.
Cattermole 2010 [116]	1370	Hong Kong	P	0 to 11 years	EPLS, Broselow tape, MAC formula	20–30%	1, 2	Low	Findings: MAC formula outperformed Broselow tape in children > 5 years. Comments: MAC actually only more accurate in children from 9 to 11 years who were too tall for the Broselow tape. Poor accuracy of all systems demonstrated. Limitations: Broselow tape not actually used, and 1998 edition values used. No evidence provided for desired accuracy targets.
Lulic 2010 [117]	209	Croatia	P	0 to 14 years	EPLS, Argall, BG, Luscombe, Broselow tape	< 10%^†^	1, 2	Low	Findings: Broselow tape was more accurate than age-based formulas but tended to slightly underestimate weight. Comments: Abstract; none of the systems estimated weight with an acceptable degree of accuracy. Limitations: Broselow tape version not reported. Incomplete presentation of data.
Rosenberg 2010 [118]	401	USA	P	0 to 14 years	healthcare provider guesses	None	1	Low	Findings: Large errors in adrenaline doses were possible, based on doctors’ guesses of weight. Comments: Abstract. Poor weight estimates translated into potentially harmful medication errors. Limitations: Incomplete presentation of data.
Williams 2010 [35]	468	Australia	P	4 and 6 years	healthcare provider guesses	None	1, 2	Low	Findings: Both age and weight were estimated poorly by paramedics, with no association with experience or training. Comments: Estimation of weight and age from images of two children by 234 estimators. Limitations: Incomplete presentation of data.
Bourdeau 2011 [119]	243	Canada	R	1 to 10 years	Broselow tape	< 10%^†^	1, 2	Low	Findings: Broselow tape was not accurate in First Nations’ children and may need to increase weight estimates by 12%. Although ideal method of dosing is still unknown, it is best practice to eliminate any possible contributing inaccuracies. Comments: First Nations’ children selected with high incidence of obesity in this population. Broselow tape did not perform equally well in all ethnic/population groups. Limitations: Incorrect use of some statistics. Broselow tape not actually used and Broselow tape version not reported.
Cattermole 2011 [45]	1248	Hong Kong	P	1 to 10 years	EPLS, ARC, Nelson, Shann, Leffler, BG, Argall, Luscombe, Theron, CAWR-1, CAWR-2	< 30%	1, 2	Low	Findings: CAWR-1 should be used in younger Chinese children, but other methods of weight estimation should be used in older children as age formulas are inaccurate. Comments: Comprehensive statistics. Findings suggested that no age formula performed well at any age. Limitations: No evidence to support suggested target of weight estimation.
Cattermole 2011 [120]	–	–	–	–	–	< 40%	1	N/A	Comments: Letter. “Age-based paediatric weight estimation is imprecise”. The author reflects that an error of ± 40% might not be considered acceptable by most clinicians, but no alternative suggested.
Costelloe 2011 [121]	62	UK	P	6 months to 6 years	Parental estimates, parent vs. nurse measurement	< 2 kg	1	High	Findings: Parents can estimate weight accurately based on home measurements, but very few actually weigh children at home. Comments: Authors used doses of ibuprofen and paracetamol as endpoints: the applicability in emergency situations is uncertain. Limitations: Incomplete presentation of data. Very small sample size.
Geduld 2011 [122]	2832	South Africa	P	0 to 10 years	EPLS, Luscombe, BG, Broselow tape	< 10%*	1, 2	Low	Findings: Broselow tape and EPLS formula most accurate in this population. Careful titration of drugs and use of clinical judgement most important in using medications safely. Comments: Data from a poor community in South Africa. The accuracy of EPLS formula was the best ever reported while the accuracy of Broselow tape was on par with other reports. Only 4% of children excluded as too tall for the tape. The authors question whether the differences in accuracy of any weight estimation system are likely to affect outcomes. Limitations: Broselow tape not actually used and Broselow tape version not reported.
Huybrechts 2011 [123]	275	Belgium	P	3 to 7 years	Parental estimates, parent vs. nurse measurement	None	1	Low	Findings: Parental estimates were most accurate when based on measurements made at home, rather than onguesses. Comments: Application to emergency weight estimations is uncertain as parental stress may negate this effect. Limitations: Targets were estimations of overweight or underweight. Incomplete presentation of data.
Kelly 2011 [124]	410	Australia	P	1 to 10 years	Luscombe, EPLS, Argall, BG	< 10%*	1, 2	Low	Findings: The Luscombe formula performed best of all the formulas. Comments: Study population BMI was 17. Same population as Nguyen 2007 and several other publications; all formulas performed poorly. Limitations: Incomplete presentation of data.
Knight 2011 [125]	657	USA	R	–	Broselow tape	None	1, 2	Low	Findings: Broselow tape performed poorly, potentially leading to under-resuscitation in all weight categories, especially in younger children. Drug doses correct in only about 50% of cases. Consensus opinion required whether to use IBW or TBW during resuscitation. Comments: Broselow tape 2007B. High incidence of obesity in study population. Limitations: Broselow tape not actually used. Incomplete presentation of data. No direct assessment of accuracy of weight estimation.
Luscombe 2011 [126]	64,197	UK	R	1 to 16 years	EPLS, Luscombe	None	1, 2	Low	Findings: The Luscombe formula outperformed the EPLS formula. Weight estimation is of paramount importance in resuscitation, therefore remembering one formula better than several. Comments: While the bias of the Luscombe formula was smaller, both formulas performed poorly. Limitations: Incomplete presentation of data. An inappropriate age range was used for formulas (up to 16 years).
Marlow 2011 [27]	140,314	UK	R	0 to 16 years	EPLS, Argall, Luscombe, BG	< 10%*	1, 2	Low	Findings: The EPLS formula was least accurate of commonly used formulas. The BG and Luscombe formulas were very similar and the best performers. No formulas showed acceptable accuracy, however. Comments: Abstract, with additional data supplied by author. This was a very large retrospective database study with good descriptive statistics. Limitations: Some incomplete data.
Rosenberg 2011 [38]	372	USA	P	0 to 14 years	healthcare provider guesses, Broselow tape	< 10%*	1, 2	Low	Findings: The Broselow tape was better than guesses by doctors, but not in obese children. Poor assessment of habitus by doctors. IBW suggested as the best target for estimation in obese kids. Comments: Broselow tape 2007B. 35% of study population obese or overweight. Mean BMI was 17.4. It is a reflection of how poorly the Broselow tape performed in obese children that doctor estimates were better; overall findings of Broselow tape accuracy similar to other studies. Limitations: Incomplete data presentation. Incorrect use of some statistics.
Wells 2011 [127]	–	–	–	–	–	< 10%*	1	N/A	Findings: More than 50% of study population required habitus-modified weight estimation. Length-based systems should be used for weight estimation in emergencies. Comments: Editorial comment. Insufficient evidence exists to choose between IBW and TBW for drug dose calculations. Limitations: No reference standard for weight estimation included.
Abdel-Rahman 2012 [15]	1938	USA	R	0 to 16 years	EPLS, ARC, Argall, BG, Broselow tape, Cattermole, Leffler, Luscombe, Nelson, Shann, TJ, TK, Mercy method	None	1, 2	Low	Findings: The Mercy method performed significantly better than the age-based, length-based and MAC-based systems. Comments: This was the original development and validation study of the Mercy method. Good mix of overweight children, but few underweight kids in sample. Broselow tape unable to be used in more than one third of the sample (too tall for the tape). Limitations: Broselow tape not actually used and Broselow tape version not reported.
Ali 2012 [128]	1723	Trinidad	R	1 to 5 years	EPLS, Luscombe, new formula	< 10%^†^	1, 2	Low	Findings: All formulas performed similarly and all poorly, even the new formula derived from the study population. Comments: Only children aged 1 to 5 are included in study. Limitations: No validation sample for derived formula. Incomplete presentation of data.
Cantle 2012 [129]	2253	USA	R	1 to 10 years	EPLS, new APLS, CAWR-1, CAWR-2, MAC	None	1, 2	Low	Findings: CAWR-2 performed as well as other formulas. Age formulas performed less well in older children. MAC performed well in older children but poorly in younger children. Comments: Conference presentation. Study of CAWR in a Western population. No age formula actually performed well in any age group. Limitations: Some errors in presented data and calculations.
Elgie 2012 [130]	188	UK	P	10 weeks to 10 years	APLS, EPLS, Luscombe formulas based on clothing label age	None	1	High	Findings: Using the age on clothing label was more accurate than using actual age. Comments: This clever study used clothing size as a surrogate marker for habitus. The Luscombe formula performed slightly better than the EPLS formula. Limitations: Incomplete presentation of data.
Garwood 2012 [131]	1252	UK	R	1 to 16 years	EPLS, new formula	None	1	High	Findings: The Garwood formula was better than the EPLS formula. Comments: Abstract. Authors used assessment of mean bias only. Limitations: Limited statistical analysis made findings difficult to interpret. No validation of new formula.
Heyming 2012 [132]	491	USA	P	IQR 10 to 49 months	Broselow tape EMS vs. Broselow tape ED	None	1	High	Findings: The Broselow tape was an accurate tool to estimate weight. Comments: Broselow tape agreement between pre-hospital personnel and ED personnel only 70.1%. Limitations: Most of study population < 4 years. Incomplete presentation of ldata. Broselow tape version not reported.
Kelly 2012 [26]	–	–	–	–	–	None	1	N/A	Findings: Best systems to be used, in order of accuracy: parental estimates, Broselow tape, age-based formulas. Comments: Book chapter. Limitations: No mention of acceptable targets for weight estimation or weaknesses of age formulas. Subjective assessment of studies only.
Meguerdician 2012 [2]	–	–	–	–	–	None	1	N/A	Findings: The Broselow tape is the most consistent and reliable tool for weight estimation, but habitus-based weight adjustment for Broselow tape is logical. Parental estimates may rival the Broselow tape, but parents may be absent or uncertain, especially under stress. Underdosing might be prudent in emergencies. Comments: The author acknowledges that the degree of acceptable error is difficult to define. Limitations: No evidence provided of efficacy of Broselow tape in reducing medication errors. No suggestion of goal target accuracy required. Some controversial opinions about drug dosing.
Milne 2012 [33]	6361	Canada	R	IQR 1.6 to 7.7 years	Broselow tape	< 30%	1, 2	Low	Findings: Broselow tape effective, although significantly underestimated weight. Similar findings in urban and rural children. Comments: Broselow tape edition 2002A. The authors suggested that an error range of 30% is reasonable and that, ideally, technology should be developed to estimate weight. Limitations: Broselow tape not actually used—data taken from anthropometric measurements.
Park 2012 [28]	124,095	Korea	R	0 to 14 years	EPLS, Shann, Leffler, Nelson, BG, Broselow tape, Park	None	1, 2	Low	Findings: All methods underestimated weight, possibly because of a secular trend towards increasing BMI. The Park formula performed best and was accurate in children < 1 year of age. Comments: All methods assessed showed poor accuracy. Limitations: Incomplete presentation of data. Poor interpretation of bias vs. accuracy. Broselow tape was not actually used and Broselow tape version not reported.
Seddon 2012 [133]	599	UK	P	1 month to 12 years	EPLS, new APLS, Argall, BG, Luscombe	None	1, 2	Low	Findings: Mixed racial study population, no major racial differences found in performance of formulas. There was increased underestimation of weight with increasing age. The Luscombe formula performed better than EPLS and APLS in children 1 to 10 years of age; BG and APLS better outside these ages. Limitations: Limited statistical analysis and incomplete data—only measures of bias reported. Some data values were not credible.
Sinha 2012 [134]	118	USA	P	0 to 14 years	Broselow tape	None	1, 2	Low	Findings: It was possible to weigh children during trauma resuscitation. Comments: Broselow tape weight was compared to stretcher weight. Nearly 40% of children in the study could not be weighed—more often the sicker kids (not fully explained). There was no validation of the accuracy of weight measured during resuscitation. Poor performance by Broselow tape with 18% of children too tall for the tape. Limitations: Incomplete presentation of data. Broselow tape version not reported.
Trakulsrichai 2012 [135]	595	Thailand	P	0 to 12 years	Broselow tape, parental estimates, growth charts	< 10%^†^	1, 2	Low	Findings: Family member estimation was most accurate, and the Broselow tape the most accurate of other weight estimation methods. Comments: Equal underestimation and overestimation by Broselow tape while family estimates tended to overestimate. Limitations: Incomplete presentation of data. Broselow tape version not reported.
Winship 2012 [42]	–	–	–	–	–	None	1	N/A	Findings: As average weight of children has increased so the accuracy of formulas has decreased; age-based methods will be unable to adjust to deal with future rises in average weights of children; no age-based weight formula accurate—might as well continue with EPLS formula. Comments: Semi-systematic review. Limitations: No suggestion of goal target accuracy required.
Wozniak 2012 [136]	777	Botswana	P	18 months to 12 years	EPLS, Luscombe, Theron, Cattermole, Broselow tape	< 10%^†^	1, 2	Low	Findings: Prediction models incorporating MAC and either tibia or ulna length performed extremely well. Age-based formulas were very inaccurate. Comments: Masters dissertation. Weight was markedly overestimated by formulas in this population with a high prevalence of HIV. Limitations: Incomplete statistics.
Abdel-Rahman 2013 [18]	624	USA	P	0 to 16 years	Mercy method two-dimensional, 3D, Broselow tape	None	1, 2	Low	Findings: Both two-dimensional and 3D Mercy methods outperformed the Broselow tape. Comments: Study population BMI was 17.3. Children much older than most weight-estimation studies included. Multicentre study—3 in the USA. One third of children were too tall for the tape. The 3D tape was significantly less accurate than the two-dimensional method. Limitations: Broselow tape not actually used and Broselow tape version not reported.
Abdel-Rahman 2013 [19]	976	USA	P	0 to 16 years	Mercy method, EPLS, Broselow tape, Luscombe, Nelson	< 20%	1, 2	Low	Findings: The Mercy method was accurate across a wider age range than other methods. Comments: Study population BMI was 17.6 with very few underweight children. Inter-rater assessment was generally reasonable, but two of the raters were inferior to the others. Limitations: Broselow tape not actually used and Broselow tape version not reported.
Akabarian 2013 [137]	403	Iran	P	0 to 14 years	Broselow tape, parental estimates	< 10%*	1, 2	Low	Findings: The Broselow tape was more accurate than parental estimates. Comments: Article in Arabic. Very good performance of Broselow tape compared to previous studies. Limitations: Exclusion criterion of weight > 35 kg limited the assessment of Broselow tape accuracy. Limited statistical analysis and data presentation. Broselow tape version not reported.
Cattermole 2013 [138]	171	Hong Kong	P	7 to 11 years	Broselow tape	None	1	High	Findings: Children “too tall for the tape” do not have full adult weight—this assumption would lead to an average 30% overestimation of weight in this study. About 40% of children were too tall for the tape. Comments: Selective evaluation of children who were too tall for Broselow tape. Interesting data which highlights a flaw in the Broselow tape methodology. Broselow tape was not practically useful over the age of 10. An overestimation of 30% would be unacceptable; no mention of desirable target. Limitations: Relatively small sample size.
Graves 2013 [139]	37,114	USA	R	0 to 14 years	EPLS, new APLS, BG, Luscombe, Broselow tape	< 10%^†^	1, 2	Low	Findings: New APLS formula was best for infants, BG best for older children. Broselow tape wrong zone in up to 60% of children. Comments: Broselow tape 2007B. Despite criticisms of Broselow tape, it outperformed the formulas in every analysis. This study had some of the poorest performances of aged-based formulas in any study. A post hoc study population BMI was calculated to be 18 to 22. Limitations: Incomplete presentation of data. Broselow tape not actually used.
Hegazy 2013 [140]	508	Egypt	P	1 to 16 years	EPLS, Shann, Garwood formula	< 10%^†^	1, 2	Low	Findings: Garwood formula performed best, especially in older children. Comments: Sample population of cancer patients. Very poor performance of all formulas tested—none were close to acceptable accuracy. Limitations: Formulas used for children older than intended. Poor interpretation of findings.
House 2013 [141]	967	Kenya	P	0 to 14 years	Broselow tape, EPLS, Nelson	MPE < 10%	1, 2	Low	Findings: Broselow tape performed better than formulas and a measure of habitus assessment (e.g. MAC) was suggested. Broselow tape should be used rather than formulas. Comments: Broselow tape 2007B. Underestimation of weight predominated. Limitations: Incomplete statistical analysis and data presentation. Poor interpretation of results from previous studies; flawed outcome measures used (indicators of bias only).
Lim 2013 [39]	199	USA	R	0 to 17 years	healthcare provider guesses	< 20%^†^	1, 2	Low	Findings: The authors suggested that EMS personnel were generally accurate in estimating weights of children. Comments: The more severe the condition, the worse the weight estimation. Weight estimation appeared better than it actually was. Limitations: Incomplete presentation of data.
Loo 2013 [142]	875	Singapore	P	1 to 10 years	EPLS, Luscombe, Broselow tape	< 10%^†^	1, 2	Low	Findings: Broselow tape more accurate than formulas. EPLS underestimated, Luscombe overestimated weight, but EPLS performed better overall than Luscombe. Comments: Broselow tape 2007B. Mean BMI of study population was 15.9. Good statistics. Limitations: Broselow tape not actually used.
Suh 2013 [143]	105,072	Korea	R	–	Broselow tape 2005A, 2007B, 2011A	< 10%*	1, 2	Low	Findings: Broselow tape 2011A more accurate than older versions. Comments: Abstract. Large database sample. Limitations: Incomplete presentation of data. Broselow tape not actually used.
Wells 2013 [5]	453	South Africa	P	0 to 12 years	Broselow tape, PAWPER tape	< 10%^†^	1, 2	Low	Findings: PT performed better than Broselow tape in every category analysed and better than any previously published system. Comments: Broselow tape 2007B. Population with mixed under- and overweight. Multi-centre study of habitus-modified length-based weight estimation. Limitations: Assessment of body habitus based on visual estimation.
Young 2013 [44]	207	USA	P	1 to 9 years	EPLS, Broselow tape, parental estimates, Luscombe, finger counting	< 10%*	1, 2	Low	Findings: Finger counting system as accurate as Broselow tape and more accurate than other formulas. Conceptually a simple system. To increase the accuracy weight-estimation systems may cause increased complexity and stress during resuscitations. Comments: The finger counting method is equivalent to formula Wt = 2.5 × age(years) + 7.5. Median BMI of study population was 17.2. Limitations: Incomplete presentation of data. Broselow tape not actually used and version not reported.
Abdel-Rahman 2014 [4]	–	–	–	–	–	None	1	N/A	Findings: Some experts have suggested that weight estimation cannot be accurate. This is likely to be to the disadvantage of children as dual length-and habitus-based can achieve acceptable accuracy. Comments: Brief review of weight estimation systems. Limitations: No target of weight estimation accuracy suggested; Mercy method recommended for environments where no scale available—no mention of emergency use.
Ackwerh 2014 [144]	10,488	USA	R	2 to 12 years	EPLS, Luscombe, Theron	< 10%*	1	High	Findings: Unreliable. Comments: Unclear statistical analysis. The reported results for age-based formulas were the best ever reported, but are completely incorrect. The graphically presented data contradict the other findings. Limitations: Incomplete presentation of data.
Allison 2014 [54]	2102	Australia	R	0 to 5 years	EPLS, BG, Luscombe, Argall, Nelson, Broselow tape, Sandell tape	None	1, 2	Low	Findings: Broselow tape was most accurate in this study. Comments: Broselow tape 2007 edition B. Aboriginal and Torres Strait Island children included (low weight-for-length). Only published study of Sandell tape. Limitations: Broselow tape not actually used. Incomplete presentation of data. Narrow age range evaluated. Very fat and very thin children excluded from study.
Batmanabane 2014 [49]	374	India	P	0 to 16 years	EPLS, ARC, Argall, BG, Broselow tape, Cattermole, Leffler, Luscombe, Nelson, Shann, TJ, TK, Mercy method	None	1, 2	Low	Findings: Mercy method performed well in Indian children, similar to that shown in Western populations. Comments: Good statistics. Overestimation of weight by all methods except Mercy. Limitations: Broselow tape not actually used and Broselow tape version not reported.
Chiengkriwate 2014 [145]	3869	Thailand	R	0 to 15 years	Broselow tape	< 10%*	1, 2	Low	Findings: Broselow tape underestimated weight in Thai children, more so in older children, similar to findings in Western populations. Comments: Broselow tape 2007 edition A. Broselow tape performance consistently at a PW10 of just below 60%. Limitations: Broselow tape not actually used.
Dicko 2014 [50]	473	Mali	P	0 to 16 years	Mercy, EPLS, ARC, Broselow tape, Nelson	None	1, 2	Low	Findings: Mercy method performed extremely well in this population in Mali, similar to its performance elsewhere in the world. Other methods overestimated weight. Comments: BMI of study population was 15.6, with 22% underweight and 1.7% overweight or obese. Good inter-rater reliability. Limitations: Broselow tape not actually used and version not reported.
Eke 2014 [146]	370	Nigeria	P	1 to 12 years	APLS	None	1	High	Findings: The APLS formula underestimated weight in these Nigerian children. Comments: The findings are unreliable in view of the major methodological flaws. Limitations: Limited and incomplete data presentation.
Erker 2014 [147]	N/A	WHO/CDC reference centiles	R	1 to 12 years	EPLS, APLS, Luscombe, BG, Park, Shann, Nelson, novel habitus-modified formulas	None	1	High	Findings: Potentially enhanced accuracy of age-based equations by using a habitus-specific formula, if length-based methods not available. Comments: Three formulas for “thick”, “normal” and “thin” children. Theoretical accuracy in derivation study. Limitations: No original data or validation of formulas. Incomplete presentation of data.
Flannigan 2014 [46]	10,081	UK	R	0 to 15 years	New APLS, Luscombe, novel formula	None	1, 2	Low	Findings: New APLS formula very inaccurate and should not be used. Weight should be adjusted according to a data table with the 5th and 95th weight-for-height values. Comments: Study performed in ICU patients. Limitations: Incomplete presentation of data. New regression formula untested, even with an internal validation sub-sample. Adjustment of weight estimation based on a “guess”.
Omisanjo 2014 [148]	2754	Nigeria	P	1 month to 11 years	Best Guess, Nelson	MPE < ±5%	1, 2	Low	Findings: Neither formula was accurate in Nigerian children with a substantial overestimation of weight. Comments: This was one of the largest prospective studies of age-based formulas in the developing world. Limitations: Some limitations from incomplete statistics.
Abdel-Rahman 2015 [64]	400**	USA	P	2 month to 16 years	EPLS, Luscombe, DWEM, HCP guess, Broselow tape, 2DMT, 3DMT		1	High	Main results: “Real world” performance of weight estimation less accurate than when performed by experts. Calculation errors were common with age-based formulas. Weight and habitus were often underestimated by visual inspection. Usage errors with Broselow tape and Mercy tapes were common. Limitations: Not all modern weight estimation systems evaluated.
Asskaryar 2015 [57]	1185	India	P	1 month to 12 years	Broselow tape	< 10%^†^	1, 2	Low	Findings: Broselow tape significantly overestimated weight in Indian children. An 8% modification of the tape improved its accuracy. Comments: Broselow tape 2007 edition B. The improved performance was not substantially better than the original and was still below acceptable performance. Recalibrating bias alone is not enough when precision is low. Limitations: Incomplete presentation of data.
Badeli 2015 [149]	216	Iran	P	1 to 10 years	DWEM, Oakley, TJ, TK, MAC, Theron, Leffler, EPLS, healthcare provider guess, parental estimate	None	1	High	Findings: The authors reported that healthcare provider guesses and EPLS formula were more accurate than other methods. Comments: The findings are completely unreliable and the conclusions consequently unreasonable. Limitations: Incomplete presentation of data. Analysis of bias confused with accuracy.
Britnell 2015 [47]	376	New Zealand	P	5 to 10 years	EPLS, Shann, Theron, Broselow tape	< 10%^†^	1, 2	Low	Findings: Broselow tape more accurate than age-based formulas in children < 143 cm. Current acceptance of formulas needs to change. Large differences in accuracy of weight estimation in different ethnic groups. Comments: Broselow tape 2011 edition A. Large proportion of Pacific Island children in sample. Broselow tape could not be used in one fifth of children, but best ever performance of the Broselow tape in a study. Habitus was assessed but data not presented. Limitations: Incomplete presentation of data.
Chavez 2015 [13]	324	USA	P	1 month to 12 years	Broselow tape, PAWPER, APLS, MAC	None	1, 2	Low	Findings: Age-based formulas and the MAC formulas performed badly. The Broselow tape performed better and the PAWPER was most accurate overall, although not as accurate as in previous studies. Comments: Underestimation of obesity (and habitus score) caused underestimation of weight. High level of obesity in study population. Limitations: Broselow tape version not reported. Incomplete presentation of data. PW5 mistaken for PW10 by authors—confusing for readers.
Garcia 2015 [65]	1698	USA	P	–	PAWPER	None	1, 2	Low	Findings: The PAWPER did not perform as well as the original study. Comments: Most assignment of habitus score by nurses. Very high proportion of obesity. Limitations: Many incorrect assignments of habitus score. Children not fully undressed to assess habitus.
Khouli 2015 [150]	815	Mexico	P	0 to 12 years	Broselow tape	None	1, 2	Low	Findings: Broselow tape not accurate in Mexican population. Comments: Reasonable statistics. Both under- and overestimation found to be a problem. Nearly half of children had at least one colour zone error. Limitations: Broselow tape not actually used and Broselow tape version not reported.
Skrobo 2015 [151]	3155	Ireland	R	1 to 15 years	EPLS, Luscombe	< 15%	1, 2	Low	Main results: The Luscombe formula underestimated weight less than the EPLS formula. Comments: Increase in children’s weight in “modern” populations related to increase in both lean body weight and adipose. Limitations: Only data on bias presented. Limited and incomplete data presentation.
Talib 2015 [152]	318	USA	P	0 to 18 years	APLS, Broselow tape, MAC formula, Mercy method	None	1	High	Main results: Mercy method performed better than Broselow tape and age-based formulas in children with Down syndrome. Comments: No system performed well in this population. Limitations: Limited and incomplete data presentation.
Young 2015 [153]	207	Philippines	P	1 to 9 years	EPLS, APLS, Luscombe, BG, finger counting, Broselow tape	None	1	High	Findings: Broselow tape performed best in this population, updated APLS formula performed worst. Comments: Broselow tape 2011 edition A. Limitations: Broselow tape not actually used. Incomplete presentation of data.
AlHarbi 2016 [154]	3537	Saudi Arabia	P	1 month to 12 years	Broselow tape 2007B and Broselow tape 2011A	None	1	High	Findings: Broselow tape 2011A performed better than Broselow tape 2007B in this population. The authors suggested that the tapes were accurate. Comments: The method of statistical analysis does not support the conclusions drawn in this paper. Limitations: Unclear if the Broselow tape was used or if derived from length measurements. Limited and incomplete data presentation.
Aliyu 2016 [155]	300	Nigeria	P	0 to 5 years	Broselow tape, APLS	None	1, 2	Low	Findings: Broselow tape and APLS formulas performed well in this population. Comments: Contrary to the authors’ conclusions, although the Broselow tape and APLS formula performed similarly, they were both inaccurate. Limitations: Not clear if Broselow tape used or if derived from length measurements. Broselow tape edition not reported. Limited and incomplete data presentation.
Britnell 2016 [51]	376	New Zealand	P	5 to 10 years	Novel weight lookup table	< 10%	1	Low	Main results: Length- and habitus-based model better than age- and habitus-based model. Model outcome similar to Broselow tape. Comments: Useful additional information on value of habitus-based methods of adjusting weight estimations. Limitations: Limited and incomplete data presentation.
Clark 2016 [62]	583	Sudan	R	6 months to 5 years	Broselow tape	< 10%**	1,2	Low	Findings: The Broselow tape performed very poorly. Comments: Abstract. Study in South Sudan, the “hungriest place on earth” where 61% of study population was malnourished. There was up to a two colour-zone overestimation in severely malnourished children with only 26% agreement in normally nourished children. Very poor performance of the Broselow tape. Dangerous overestimation of weight in undernourished children. Limitations: Broselow tape not actually used and Broselow tape version not reported.
Carasco 2016 [156]	–	–	–	–	–	None	1	N/A	Main results: EPLS formula may be better suited to identifying ideal body weight rather than total body weight. Comments: Systematic review. Ideal body weight should only be used in obese children. Limitations: No weight estimation data.
Chassee 2016 [157]	197	USA	R	0 to 12 years	911 caller weight estimation	< 20%	1	Low	Main results: Family members could frequently provide a weight estimate to the 911 operator. Comments: More than 20% of cases had a weight estimation error > 20%. Limitations: Limited and incomplete data presentation.
Georgoulas 2016 [21]	300	South Africa	P	1 month to 12 years	Broselow tape, PAWPER, Wozniak, Mercy	< 10%*	1, 2	Low	Findings: PAWPER tape performed best, but good performances from Wozniak and Mercy methods. Broselow tape was worst performer. Comments: Unpublished data. Broselow tape 2011 edition A. Poor population with high proportion of underweight children. First comparative study of these methodologies. Relatively weaker performance by all methods in infants, but Wozniak especially was very weak. Limitations: Assessment of body habitus done by single researcher.
Jung 2016 [16]	906	Korea	P	0 to 17 years	Broselow tape, novel device	None	1, 2	Low	Main results: The novel device performed better than the Broselow tape in all outcome measures and was quicker to use. Comments: Both devices underestimated weights. Overweight and underweight children were often misclassified into wrong habitus category. Broselow tape 2011 edition A. Limitations: Device is not commercially available. Incomplete statistical analysis
Jung 2016 [158]	–	–	–	–	–	None	1	N/A	Non-systematic review. Main results: Weight frequently underestimated in older children. Differences in body habitus account for this error, which needs further research. Comments: Article in Korean. Limitations: Non-systematic review. No quantitative assessment of methodologies.
Lowe 2016 [55]	3018	USA	R	0 to 13 years	Broselow tape, Handtevy tape	None	1, 2	Low	Main results: The Broselow tape performed better than the Handtevy tape in all outcome measures. Both tapes underestimated weights. Comments: Although the authors recognised the increased accuracy when obese children were excluded, they still erroneously advocated “recalibration” of the tapes. Broselow tape 2011 edition A. Limitations: Tapes not actually used. Limited and incomplete data presentation.
Mishra 2016 [159]	603	India	P	0 to 10 years	Broselow tape	None	1	Low	Findings: Broselow tape performed best in smallest children. Comments: Broselow tape 2007 edition B. Only colour zone accuracy was assessed, which was poor in children > 18 kg. Limitations: Limited and incomplete data presentation.
Nosaka 2016 [160]	237	Japan	P	0 to 10 years	EPLS, Park, Broselow tape, parental estimates	None	1, 2	High	Main results: Parental estimates were most accurate, then Broselow tape then APLS formula. Mothers’ weights, as well as Broselow tape and EPLS estimations were extremely accurate. Comments: The BMI for this population suggests that very few obese children were included and the sample was skewed towards very young children. Broselow tape 2007 edition B. Limitations: Limited and incomplete data presentation.
Ralston 2016 [29]	453,990	Multicentre International	R	6 months to 5 years	Broselow tape, MAC, height + MAC model	None	1, 2	Low	Findings: A novel model incorporating height and MAC was the most accurate. Broselow tape 2011 edition A less accurate than 2007 edition B in this population. Comments: Broselow tape 2007 edition B and 2011 edition A. A very large multinational database study. Limitations: Broselow tape not actually used.
Sahar 2016 [161]	1163	Malaysia	P	0 to 12 years	Broselow tape	None	1, 2	Low	Findings: Broselow tape underestimated weight in small children and overestimated weight in older children. It was not accurate. Comments: As with studies elsewhere there was a large variation in accuracy. Limitations: Broselow tape version not reported. Limited and incomplete data presentation.
So 2016 [162]	4600	Hong Kong	R	1 to 12 years	APLS, EPLS, BG, Luscombe, CAWR-1, CAWR-3	None	1, 2	Low	Main results: A new CAWR formula was more accurate than other formulas in this study. Comments: No formula was very accurate and the differences between formulas were generally small. Limitations: Data very skewed towards younger children. No inclusion of length-based methods.
So 2016 [163]	4178	Hong Kong	R	1 to 9 years	Finger counting	None	1, 2	Low	Main results: The finger-counting formula was more accurate than other formulas. Comments: Same data was used for So 2016, but with a narrower age-restriction and the new finger-counting formula. Limitations: Duplicate data. Only the data on finger counting was added.
Tanner 2016 [17]	178	USA	P	2 to 18 years	Broselow tape	None	1	High	Main results: The Broselow tape was very inaccurate in overweight and obese children, who made up more than half the sample. Habitus assessment was poor by parents and nurses, but better by the principal investigator. Comments: Using a corrective formula based on waist circumference to adjust the Broselow tape weight improved accuracy in obese children. Limitations: Broselow tape version not reported. Limited and incomplete statistical analysis. Only included children who fell within the length limitations of the tape.
Young 2016 [9]	–	–	–	–	–	–	1	N/A	Systematic review. Main results: Parental estimates were the most accurate technique followed by length- and habitus-based methods. Broselow tape more accurate than age formulas. Limitations: No quantitative data analysis.
Bowen 2017 [164]	1381	Zambia	P	0 to 14 years	Broselow tape, APLS, EPLS, ARC, Argall, BG, CAWR, Garwood, Leffler, Luscombe, Michigan, Nelson, Park, Shann, Theron	< 10%*	1, 2	Low	Findings: Broselow tape performed better than every formula in this population, BG and Michigan formulas performed worst. Comments: None of the methods were accurate, and all methods overestimated weight. Limitations: Broselow tape not actually used. Broselow tape version not reported.
O’Leary 2017 [22]	199	Australia	P	0 to 14 years	PAWPER, APLS, Luscombe, BG, Broselow tape, Mercy	< 10%*	1, 2	Low	Findings: Age-based formulas performed badly. The Broselow tape and Mercy method performed significantly better and the PAWPER was most accurate overall. Comments: All systems performed worst in infants. Reasonably good performance of Broselow tape, possibly because of newer edition used. Limitations: Broselow tape version not reported.
Reilly 2017 [165]						None	1	N/A	Commentary. Main results: MAC is useful as an adjunct to estimate weight. Limitations: No original data.
Samerchua 2017 [68]	430	Thailand	P	0 to 12 years	Parental estimations, Mercy method, Broselow tape, EPLS, APLS	None	1, 2	Low	Main results: Parental estimates were most accurate, followed by the Mercy method. Comments: Parents could only provide an estimate in 80% of cases—the Mercy method was the only method that could be used in all children. Limitations: It was not clear what training the raters had received.
Trainarongsakul 2017 [166]	345	Thailand	P	0 to 15 years	RAMA Ped card	None	1	Low	Main results: RAMA Ped card had a fair correlation with weight. Comments: The use of only correlation data made the findings hard to interpret. Limitations: Limited and incomplete data presentation.
Waseem 2017 [167]	538	USA	R	0 to 8 years	Broselow tape	None	1	N/A	Main results: Broselow tape underestimated weight in older children. It was not accurate in nearly half the population. Broselow tape 2011 edition A. Comments: Many underweight and obese children included which cause the poor performance. Limitations: Broselow tape not actually used. Limited and incomplete data presentation. Only included children who fell within the length limitations of the tape.
Wells 2017 [20]	328	South Africa	P	0 to 16 years	Broselow tape, PAWPER, Wozniak, Mercy	< 10%^†^	1, 2	Low	Findings: The Broselow tape performed poorly in this study, the Wozniak method and the Mercy method showed intermediate accuracy and the PAWPER was most accurate overall. Comments: This was a population with many older children and children with deviations from “average” weight-for-length. The PAWPER XL tape worked well in this population. Limitations: The Mercy method was used in a simulated resuscitation setting (supine children), which may have affected its accuracy.
Wells 2017 [11]	1085	South Africa	P	0 to 16 years	20 age-based formulas, Traub-Johnson and Traub-Kichen formulas including habitus-modified age- and length-based formulas	< 10%	1, 2	Low	Findings: No age- or length-based formula performed acceptably well. Age-based habitus-modified formulas also performed poorly, but length-based habitus modified formulas were, surprisingly, extremely accurate. Comments: Length-based formulas can predict both TBW and IBW, but are mathematically complex. Limitations: Habitus assessment by visual estimation is subjective.
Wells 2017 [31]	13,134	USA	R	0 to 18 years	PAWPER XL-MAC method	< 10%	1, 2	Low	Findings: The PAWPER XL-MAC method was very accurate in data from both the USA and South Africa. Comments: This sytem was completely objective with no visual assessment of habitus. Limitations: Retrospective study
Whitfield 2017 [14]	–	–	–	–	–	–	–	–	As for Wozniak 2012

A brief summary of findings as well as a short commentary on significant aspects is included. In the description of target accuracy, some studies used an implied target (indicated by an asterisk (*)) and some expressed a clear, strong opinion (indicated by a dagger ( ^†^ )). Whether studies were entered into arm 1 (qualitative arm) only, or both arm 1 and arm 2 (meta-analysis) is indicated. The final assessment of the risk of bias is also indicated. Abbreviations: *APLS* Advanced Paediatric Life Support formula, *ARC* Australian Resuscitation Council formula, *BG* Best Guess formula, *BT* Broselow tape, *CAWR* Chinese age-weight rule formula, *DWEM* devised weight-estimating method, *EPLS* European Paediatric Life Support formula, *TJ* Traub-Johnson formula, *TK* Traub-Kichen formula

### Benchmark accuracy for a weight estimation system

After studying the 150 identified articles, only three articles were found to propose a statistically meaningful target for a weight estimation system: one article recommended that 95% of weight estimates must fall within 20% of actual weight and two articles suggested that 70% of estimates must be within 10% of actual weight *and* 95% of weight estimates must fall within 20% of actual weight [11, 30, 31]. There was, however, no evidence found upon which to base any specific measurement analysis metric for a weight estimation system. There was also no credible evidence found of a tolerable weight estimation error, in terms of safety for drug dose calculation, for an individual child.

In 90/150 articles (60.0%), there was no mention at all of an appropriate target for weight estimation accuracy. In 41/150 articles (27.3%) an error of < 10% was suggested as appropriate; in 11/150 articles (7.3%) an error of < 20% was advocated; in 2/150 articles (1.3%) an error of < 30%; and in 6/150 articles (4.0%) another value or a statistically inappropriate measure was proposed. None of the studies included any evidence to support these target figures. The values were selected based on clinical significance, pragmatic limits based on generalised therapeutic ratios, or based on guidelines on determining drug bioequivalence [32, 33].

### Meta-analysis data on bias (trueness), precision and accuracy of paediatric weight estimation systems

Table 2 contains a description of each of the weight estimation systems reviewed, as well as any restrictions on their use. The raw data and outcomes for each of the weight-estimation methodologies included in the meta-analysis are shown in Additional file 1: Table S1. From the individual study data, it could be seen that there was very poor within-study precision for most weight estimation systems (shown by the wide limits of agreement), with the exception of the two-dimensional methods, which generally had precision limits of agreement of less than ± 20%.

**Table 2:** Summary and description of weight estimation methodologies described in the literature

	Name	Formula	Restrictions/limitations/acceptable accuracy benefits	
Age-based and length-based formulas	Ali formula	*Wt* = (2.5 × *Z*) + 8	Derived in a Trinidadian population of children ≤ 5 years of age in 2012. No validation studies to date. Age restriction 1 to 5 years of age.	
	Argall formula	*Wt* = (3 × *Z*) + 6 *or* [*Wt* = 3 × (*Z* + 2)]	Developed from a small UK study in 2003 (300 children). Generally found to underestimate weight, more so in older and heavier children. Age restriction 1 to 10 years of age.	
	Advanced Paediatric Life Support formula (APLS) (new)	$Wt=\frac{z}{2}+4$	For infants ≤ 12 months of age	Derived in a UK population and adopted in 2011 by the Advanced Life Support Group from a combination of the original APLS and the Luscombe formulas. It was untested and unvalidated at the time of adoption. Generally overestimates weight. Age restriction birth to 12 years of age.
		*Wt* = (2 × *Z*) + 8 *or* [*Wt* = 2 × (*Z* + 4)]	For children aged 1 to 5 years	
		*Wt* = (3 × *Z*) + 7	For children aged 6 to 12 years	
	Australian Resuscitation Council formula (ARC)	*Wt* = 3.5	At birth	Adopted by the ARC in Australia in 1996. Same as New Zealand Resuscitation Council formula. Generally underestimates weight, more so in older and heavier children. Differing accuracy in different ethnic, socio-economic and international populations. No specific age restriction noted.
		*Wt* = (2 × *Z*) + 8	For children aged 1 to 9 years	
		*Wt* = 3.3 × *Z*	For children 10 years and over	
	Best Guess formulas (BG)	$Wt=\frac{z+9}{2}$	For infants ≤ 12 months of age	Also known as the Tinning formulas. Derived in Australian population in 2007 from a retrospective database study of more than 70,000 children. Generally overestimates weight, especially in poorer populations. Has been evaluated in several validation studies with mixed results.
		*Wt* = (2 × *Z*) + 10 *or* [*Wt* = 2 × (*Z* + 5)]	For children aged 1 to 5 years	
		*Wt* = 4 × *Z*	For children aged 6 to 14 years	
	Bicer formula	*Wt* = (3 × *Z*) + 6 *or* [*Wt* = 3 × (*Z* + 2)]	For children aged 3 to 6 years	Although these formulas are mentioned and evaluated in the Bicer study, the analysis and reporting is fatally flawed and cannot be evaluated; the origin of the first formula of the set is the same as the Argall formula. Age restriction proposed by Bicer to be 3 to 18 years.
		*Wt* = (4 × *Z*) − 3	For children aged 7 to 18 years	
	Chinese age-weight rule 1 (CAWR-1)	*Wt* = (3 × *Z*) + 5	For children aged 1 to 10 years	Developed in Hong Kong for ethnic Chinese children in 2011 from a sample of 1248 children. Age restriction 1 to 10 years (although developers advise use with caution over 7 years).
	Chinese age-weight rule 2 (CAWR-2)	$Wt=\frac{\left(Z\times 7\right)+25}{3}$	For children aged 1 to 6 years	
		*Wt* = (4 × *Z*) − 4	For children aged 7 to 10 years	
	European Paediatric Life Support formula (old APLS formula) (EPLS)	*Wt* = 2 × (*Z* + 4) *or* [*Wt* = (2 × *Z*) + 8]	Original population and date of derivation unclear. Generally underestimates weight, more so in older and heavier children. Differing accuracy in different ethnic, socio-economic and international populations. Age restriction 1 to 10 years of age	
	Garwood formula	$Wt=\frac{z}{4}+6$	Developed in a UK population from a sample of 1252 children in 2012. The initial validation study was fatally flawed, but this formula has been subjected to a validation study subsequently (showing poor performance). For children aged 1 to 16 years.	
	Leffler formulas	$Wt=\frac{z+8}{2}$	For children <1 year of age	Also known as the Tintinalli formula, the original origin is unclear, but became popular after the Leffler study in 1997. Overestimates weight in younger children (≤ 6 years) and underestimates weight in older children (> 6 years).
		*Wt* = (2 × *Z*) + 10	For children aged 1 to 10 years	
	Luscombe formula	*Wt* = (3 × *Z*) + 7	Developed in the UK in 2007 from a large database of nearly 14,000 children. Underestimates weight in most populations studied, but significantly overestimates weight in populations from developing countries. Age restriction 1 to 10 years.	
	Michigan formula	*Wt* = (3 × *Z*) + 10	Derived by Ackwerh in 2010, but has not been evaluated fully.	
	Nelson formulas (originally Weech’s formulas)	$Wt=\frac{z+9}{2}$	For infants 3 to 12 months	As described in Nelson’s Textbook of Paediatrics. The origin is probably from Weech’s formulas, first reported in 1954 in the USA. The Weech formula is still in use today as one of the standard measurement denominators for determining underweight status. Weight most often overestimated in infants and older children (> 6 years) and underestimated in younger children (≤ 6 years).
		*Wt* = 2 × (*Z* + 4)	For children aged 1 to 6 years	
		$Wt=\frac{\left(Z\times 7\right)-5}{2}$	For children aged 7 to 12 years	
	Park formulas	$Wt=\frac{z+9}{2}$	For infants ≤12 months of age	Derived in Korean population from a large database study (nearly 125,000 children). Poor accuracy in older children (> 6 years).
		*Wt* = (2 × *Z*) + 9	For children aged 1 to 4 years	
		*Wt* = (4 × *Z*) − 1	For children aged 5 to 14 years	
	Shann formulas	*Wt* = (2 × *Z*) + 9	For children aged 1 to 9 years	Used in Australasia primarily. Origin is unclear. Underestimates weight increasingly with increasing age.
		*Wt* = (3 × *Z*)	For children aged >9 years	
	Theron formula	*Wt* = *e* ^(0.175571 × *Z*) + 2.197099^	Derived in 2005 in New Zealand from a small study of 900 children that included a large number of Pacific Island children (high weight-for-age). The developers intended it for use in children high in the weight-for-age centiles. Age restriction 1 to 10 years. Overestimates weight in most populations.	
	Unknown	*Wt* = (3 × *Z*) + 8	This formula is mentioned in some weight-estimation studies with no reference to its origin. It is not known what restrictions apply. Mentioned in Dearlove, Bicer.	
	Traub-Johnson formula (TJ)	*Wt* = 2.05 × *e* ^0.02*X*^	Derived in 1980 from USA national growth data from 1959. This formula was used to estimate ideal body weight and adjusted body weight, which were used interchangeably. The formula was intended to estimate the 50th centile of weight-for-height. Underestimates total body weight. For children aged 1 to 18 years.	
	Traub-Kichen formula (TK)	*Wt* = 2.396 × 1.0188^*X*^	Derived in 1983 in the USA from data from more than 20,000 children in the National Centre for Health Statistics database. The formula was intended to estimate the 50th centile of weight-for-height which the developers regarded as an approximation of ideal body weight. Underestimates total body weight. For children over 74 cm and aged 1 to 17 years.	
Other length-based systems	Broselow tape (BT)	Weight estimated directly by placing tape next to child and measuring from head to heel. The estimated weight and colour zone is read off the tape.	Developed in 1985 in the USA from US growth data and first validated in a sample of just over 900 children in 1988. Several changes have been made over the years: the latest version is the 2011A edition. Underestimates weight except in populations with a high prevalence of poor nutrition. Inaccuracy increases with increasing length/weight. Increased underestimation of weight in obese and overweight individuals. Substantial number of children “too tall for the tape” but who are not at adult weight. Length restriction 46 to 143 cm. Maximum weight estimation 36 kg.	
	Blantyre tape	Weight estimated directly by placing tape next to child and measuring from head to heel. The estimated weight is read off the tape.	Developed in Malawi using values 85% of the 50th centile of the American National Centre for Health Statistics weight-for-length growth charts. Validated on a sample of 729 children. The developers reported a reasonable accuracy between 4 and 16 kg, but the reporting of data was fatally flawed and is unverifiable. Length restriction of 45 to 130 cm.	
	Oakley table	Age or length is used to estimate weight from a graph.	Developed in the USA in 1988 from averaged boy-girl medians of unspecified growth charts. Overestimates weight in infants and older children (> 6 years). Age restriction 0 to 14 years and length restriction 50 to 160 cm.	
Habitus-modified systems	Erker formulas	*Wt* = (2 × *Z*) + 6	For “thin” children	Developed in 2014 in the USA using regression formulas to estimate the 5th, 50th and 95th centiles of the Centre for Disease Control weight-for-age growth charts. Has not yet been shown to be accurate.
		*Wt* = (3 × *Z*) + 6	For “normal” (average) children	
		*Wt* = (4 × *Z*) + 6	For “thick” (fat) children	
	Yamamoto formulas	*See reference 11 different logarithmic formulas.*	Developed in 2009 in the USA from a sample of 542 children. A different length-based formula is selected for one of five (under 3 years of age) or six (over 3 years of age) icons, which represent an assessment of the body habitus. The reporting of the validation against the Broselow tape is flawed and does not permit verification. This technique has not been subsequently validated.	
	Wozniak formulas	*Wt* = (1.443 × *U*) + (1.596 × *M*) − 32.963	Developed in Botswana in 2012 from a sample of 777 children with a high prevalence of HIV infection and growth retardation. Measurements of mid-arm circumference and ulna length or tibia length are used to estimate weight using the formula. The accuracy of the method decreases in children <10 kg and children > 40 kg.	
		*Wt* = (0.86 × *T*) + (1.715 × *M*) − 30.426		
	Devised weight estimating method (DWEM)	Length measured and then habitus assessed as “Slim”, “Average” or “Heavy” and weight read off a chart. A pre-marked tape was developed but is not widely available.	Developed in 1986 in the USA based on standard growth data available in 1983 and validated in a small sample of 258 children. Underestimates weight, especially in taller children. Length restriction 50 to 175 cm. Maximum weight estimation 70 kg.	
	PAWPER tape	Weight estimated directly by placing tape next to child and measuring from head to heel. A habitus score (1 to 5) is assigned to the child based on body habitus (1 = very thin, 3 = average, 5 = very fat). The estimated weight for that length and habitus score is read off the tape.	Developed in 2004 in South Africa based on WHO weight-for-length growth charts and validated on a sample of 453 children in 2013. Estimates weight uniformly across length range of tape. Performs well in children who are under- or overweight. Length restriction 43 to 153 cm. Maximum weight estimation 47 kg. The extended PAWPER tape accommodates children up to 180 cm in length, a maximum weight estimation of 116 kg and with a 7-point habitus score assessment (habitus scores 6 and 7 were added to accommodate children above the 95% centile of weight-for-length, i.e. for obese and severely obese children).	
	Mercy method (MM)	Humerus length and mid-arm circumference are measured and then used to determine “segmental weights” from a table. Specifically desgined tapes “2D” and “3D” tapes may be used which eliminates the need for a data table.	Developed in the USA from a database of 19,625 children and validated across several centres in 2012, 2013 and 2014, including in developing countries. Consistently good weight estimation across age and habitus ranges. Decreased accuracy in younger children (< 2 years).	
Other	Cattermole MAC formula	*Wt* = (*M* − 10) × 3	A mid-arm circumference-based formula developed in Hong Kong ethnic Chinese children in 2010 from a sample of 1370 children. Decreased accuracy in children < 6 years and underestimation of weight in older children. Recommended by developers to restrict use to children aged 6 to 11 years.	
	Haftel formula	*Wt* = (5.176 × *LW*) + 3.487	The “hanging-leg weight” formula developed in the USA in 1990 from a small sample of 100 anaesthetised children aged 2 months to 15 years. The accuracy of weight estimation was worst in infants and increased with age. No subsequent studies have been reported.	
	Bavdekar formula	*Wt* = − 5.15 + (*FL* × 1.35)	Developed in India in 2006 from a sample of 500 infants < 2 years of age. Fatal flaws in the methodology do not permit the interpretation of the accuracy of this formula. No subsequent studies have been reported. For infants ≤ 2 years.	

Methods not shown include the Carroll method, the Sandell and Handtevy tapes (insufficient data) and neonatal weight estimation applications (out of scope). Only weight estimation systems that had more than one article assessing their functioning were considered for inclusion into the meta-analysis. Abbreviations: *Z* age in years, *z* age in months, *X* height or length in centimetre, *M* mid-arm circumference in centimetre, *LW* hanging leg-weight in kilogramme, *FL* foot length in centimetre, *U* ulna length in centimetre, *T* tibial length in centimetre

**Fig. 2:**
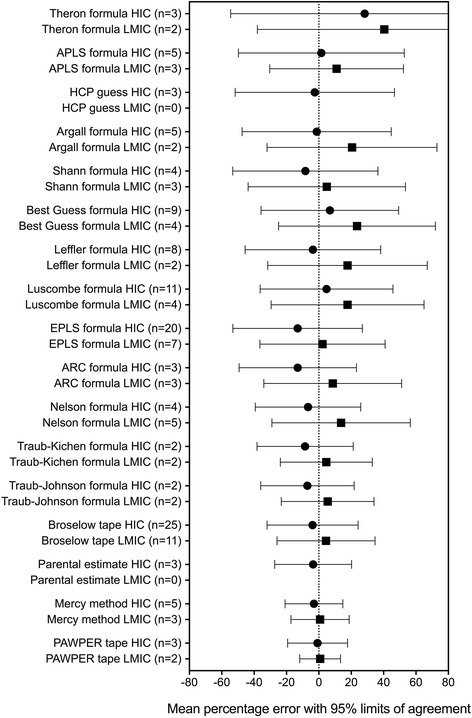
Forest plot showing the bias and precision data of the major weight estimation systems evaluated

Figure 2 shows the pooled data of the bias and precision for the weight-estimation systems evaluated. The fixed effects outcomes and data for the weight estimation methods not presented in Fig. 2 can be found in Table 3. The important findings can be summarised as follows:

- There was a wide variation in the weight estimation bias between low- and middle-income countries (overestimation) and high-income countries (underestimation). This was most noticeable with the age-based systems, less so with the length-based systems and least with the two-dimensional systems, which had virtually zero bias.
- There were very wide limits of agreement for all methods other than the PAWPER tape and the Mercy method.

**Table 3:** Weight estimation meta-analysis summary data, showing both fixed effects (FE) and random effects (RE) data

		Random effects outcomes									Fixed effects outcomes								
		MPE	LLOA	ULOA	Studies	*N*	PW10	PW20	Studies	*N*	MPE	LLOA	ULOA	Studies	*N*	PW10	PW20	Studies	*N*
APLS (new)	All	5.1	−42.5	52.6	8	19,029	33.6	56.1	13	49,709	6.1	−44.1	56.2	8	19,029	28.3	58.8	13	49,709
APLS (new)	HIC	1.5	−49.7	52.8	5	17,318	33.7	56.1	9	46,709	5.8	−44.7	56.3	5	17,318	28.0	58.8	9	46,709
APLS (new)	LMIC	10.9	−30.4	52.3	3	1711	33.4	50.6	4	3000	9.1	−37.2	55.4	3	1711	33.8	56.9	4	3000
ARC	All	−2.3	−41.6	37.1	6	6334	35.9	64.2	7	7405	−8.3	−50.5	33.8	6	6334	35.9	65.3	7	7405
ARC	HIC	−13.1	−49.3	23.2	3	4572	35.1	66.7	3	4572	−13.4	−50.1	23.2	3	4572	34.3	64.9	3	4572
ARC	LMIC	8.6	−34.0	51.1	3	1762	36.5	62.4	4	2833	4.9	−39.4	49.2	3	1762	38.6	65.9	4	2833
Argall formula	All	4.9	−43.0	52.8	7	145,823	31.5	51.1	10	147,341	−4.7	−47.3	37.9	7	145,823	38.7	63.1	10	147,341
Argall formula	HIC	−1.3	−47.4	44.7	5	144,691	34.7	54.3	7	145,257	−4.8	−47.2	37.6	5	144,691	38.9	63.2	7	145,257
Argall formula	LMIC	20.5	−32.1	73.1	2	1132	24.1	46.8	3	2084	14.3	−37.4	66.1	2	1132	28.4	54.1	3	2084
Best Guess formula	All	12.0	−32.4	56.3	13	284,798	33.8	58.2	18	324,035	5.8	−35.2	46.8	13	284,798	39.2	65.1	18	324,035
Best Guess formula	HIC	6.8	−35.7	49.3	9	277,790	37.7	63.5	14	318,478	5.5	−35.2	46.1	9	277,790	39.5	65.3	14	318,478
Best Guess formula	LMIC	23.6	−24.9	72.1	4	7008	20.2	46.4	4	5557	19.3	−25.5	64.1	4	7008	21.9	58.5	4	5557
CAWR-1	All	−3.3	−45.4	38.9	4	9064	34.6	65.7	5	10,016	−5.2	−48.2	37.7	4	9064	34.4	65.2	5	10,016
CAWR-1	HIC	−5.6	−47.1	35.9	3	8101	34.1	68.1	3	8101	−6.3	−48.6	36.0	3	8101	34.2	65.9	3	8101
CAWR-1	LMIC	3.9	−40.4	48.2	1	963	35.5	62.0	2	1915	3.9	−40.4	48.2	1	963	35.5	62.0	2	1915
CAWR-2	All	1.2	−38.6	40.9	3	4384	41.4	76.2	3	4384	−1.0	−42.1	40.0	3	4384	42.2	76.3	3	4384
CAWR-2	HIC	−2.2	−41.2	36.8	2	3501	41.7	79.0	2	3501	−3.3	−43.1	36.5	2	3501	42.6	77.7	2	3501
CAWR-2	LMIC	7.9	−33.3	49.1	1	883	40.7	70.8	1	883	7.9	−33.3	49.1	1	883	40.7	70.8	1	883
EPLS formula	All	−9.1	−48.8	30.6	27	351,449	35.9	63.0	36	190,580	−14.2	−56.1	27.7	27	351,449	33.8	56.6	36	190,580
EPLS formula	HIC	−13.1	−53.1	26.9	20	344,221	34.7	61.0	27	185,925	−14.5	−56.4	27.4	20	344,221	33.5	56.0	27	185,925
EPLS formula	LMIC	2.3	−36.4	40.9	7	7228	39.7	68.0	9	4655	−1.9	−39.1	35.2	7	7228	46.3	71.8	9	4655
Garwood formula	All	11.8	−38.9	62.5	2	1390	31.8	58.3	3	2461	10.7	−38.3	59.6	2	1390	30.6	57.3	3	2461
Garwood formula	HIC	9.2	−36.5	54.9	1	996	35.3	65.9	1	996	9.2	−36.5	54.9	1	996	35.3	65.9	1	996
Garwood formula	LMIC	14.4	−41.3	70.1	1	394	30.1	54.5	2	1465	14.4	−41.3	70.1	1	394	27.5	51.5	2	1465
Leffler formula	All	0.6	−42.8	44.0	10	270,349	32.6	57.4	11	271,373	−1.5	−40.1	37.2	10	270,349	43.8	69.3	11	271,373
Leffler formula	HIC	−3.7	−45.6	38.2	8	269,130	35.9	62.0	8	269,130	−1.5	−40.1	37.0	8	269,130	43.9	69.5	8	269,130
Leffler formula	LMIC	17.8	−31.6	67.1	2	1219	23.7	45.2	3	2243	11.8	−36.7	60.3	2	1219	27.8	52.3	3	2243
Luscombe formula	All	8.2	−34.5	50.9	15	237,568	34.0	58.1	21	275,841	0.4	−41.5	42.4	15	237,568	38.7	67.1	21	275,841
Luscombe formula	HIC	4.7	−36.3	45.8	11	231,881	38.3	64.7	15	269,554	0.2	−41.6	41.9	11	231,881	39.0	68.0	15	269,554
Luscombe formula	LMIC	17.7	−29.5	65.0	4	5687	23.1	46.2	6	6287	11.8	−33.7	57.2	4	5687	27.1	54.2	6	6287
Nelson formula	All	4.7	−33.6	42.9	9	134,315	36.8	61.9	10	135,339	−9.1	−38.6	20.5	9	134,315	42.0	73.7	10	135,339
Nelson formula	HIC	−6.7	−39.3	25.9	4	128,844	36.6	59.7	5	129,173	−9.8	−38.0	18.3	4	128,844	42.1	73.9	5	129,173
Nelson formula	LMIC	13.8	−29.0	56.5	5	5471	37.0	63.7	5	6166	8.9	−30.3	48.0	5	5471	40.9	70.1	5	6166
Park formula	All	5.9	−35.4	47.2	2	125,170	36.9	64.3	4	126,788	1.0	−34.8	36.8	2	125,170	41.5	72.4	4	126,788
Park formula	HIC	0.9	−34.8	36.6	1	124,095	43.4	74.2	2	124,332	0.9	−34.8	36.6	1	124,095	41.7	72.8	2	124,332
Park formula	LMIC	10.9	−35.9	57.7	1	1075	30.5	54.5	2	2456	10.9	−35.9	57.7	1	1075	29.4	53.0	2	2456
Shann formula	All	−2.7	−49.1	43.8	7	107,890	35.8	60.5	9	111,266	−8.5	−41.9	24.9	7	107,890	40.2	70.9	9	111,266
Shann formula	HIC	−8.3	−53.2	36.5	4	106,150	37.2	62.1	5	108,455	−8.7	−41.6	24.3	4	106,150	40.3	71.1	5	108,455
Shann formula	LMIC	4.9	−43.7	53.5	3	1740	34.1	58.9	4	2811	3.4	−45.5	52.2	3	1740	35.5	61.2	4	2811
Theron formula	All	33.2	−47.9	114.2	5	4606	17.6	38.4	8	6362	29.4	−65.2	124.0	5	4606	18.8	13.5	8	6362
Theron formula	HIC	28.4	−54.5	111.3	3	3272	20.0	44.6	4	3577	27.8	−73.0	128.6	3	3272	18.8	13.5	4	3577
Theron formula	LMIC	40.3	−38.0	118.6	2	1334	15.2	32.2	4	2785	33.3	−43.7	110.4	2	1334	18.7	13.5	4	2785
Parental estimate	All	−3.6	−27.3	20.2	3	561	69.8	87.1	10	3070	−3.2	−24.9	18.4	3	561	78.1	89.8	10	3070
Parental estimate	HIC	−3.6	−27.3	20.2	3	561	68.0	87.1	9	2475	−3.2	−24.9	18.4	3	561	76.4	89.8	9	2475
Parental estimate	LMIC				0		85.2		1	595				0		85.2	595	1	
Healthcare provider guesses	All	−2.5	−51.7	46.8	3	547	35.6	59.3	5	1719	−2.8	−49.1	43.4	3	547	40.3	58.1	5	1719
Healthcare provider guesses	HIC	−2.5	−51.7	46.8	3	547	35.6	59.3	5	1719	−2.8	−49.1	43.4	3	547	40.3	58.1	5	1719
Healthcare provider guesses	LMIC				0				0					0				0	
Finger counting	All				0		53.8	83.4	2	4385	0.1	−34.0	34.2	0	4178	49.1	80.0	2	4385
Finger counting	HIC				0		53.8	83.4	2	4385	0.1	−34.0	34.2	0	4178	49.1	80.0	2	4385
Finger counting	LMIC				0				0		0.1	−34.0	34.2	0	4178			0	
Traub-Johnson formula	All	−0.8	−29.5	27.9	4	3561	45.9	74.8	4	3561	−3.2	−34.3	27.9	4	3561	48.8	75.2	4	3561
Traub-Johnson formula	HIC	−7.1	−35.9	21.7	2	2126	46.6	74.9	2	2126	−7.1	−37.4	23.2	2	2126	45.7	71.3	2	2126
Traub-Johnson formula	LMIC	5.5	−23.2	34.1	2	1435	45.2	74.7	2	1435	2.6	−26.4	31.5	2	1435	53.4	81.1	2	1435
Traub-Kichen formula	All	−2.0	−31.0	27.1	4	3455	45.4	74.4	4	3455	−4.8	−36.5	26.8	4	3455	48.5	74.4	4	3455
Traub-Kichen formula	HIC	−8.5	−38.2	21.2	2	2121	45.5	72.9	2	2121	−9.1	−39.8	21.6	2	2121	45.4	69.6	2	2121
Traub-Kichen formula	LMIC	4.6	−23.8	33.0	2	1334	45.3	75.9	2	1334	2.0	−26.4	30.4	2	1334	53.6	82.1	2	1334
MAC formula	All	6.7	−23.3	36.6	4	458,019	29.6	52.8	6	459,120	4.6	−16.0	25.3	4	458,019	28.0	58.0	6	459,120
MAC formula	HIC	7.4	−25.8	40.5	3	4029	29.3	51.5	4	4353	9.7	−29.4	48.9	3	4029	34.0	60.4	4	4353
MAC formula	LMIC	4.6	−15.8	25.0	1	453,990	30.1	58.0	2	454,767	4.6	−15.8	25.0	1	453,990	27.9	58.0	2	454,767
DWEM	All	−1.1	−26.3	24.1	2	884	57.8	87.6	3	1142	−0.9	−26.2	24.4	2	884	57.7	88.0	3	1142
DWEM	HIC	−1.1	−26.3	24.1	2	884	57.8	87.6	3	1142	−0.9	−26.2	24.4	2	884	57.7	88.0	3	1142
DWEM	LMIC				0				0					0				0	
PAWPER tape	All	−0.2	−16.3	16.0	5	15,913	78.0	96.6	7	15,159	−1.0	−18.4	16.4	5	15,913	81.3	97.8	7	15,159
PAWPER tape	HIC	−0.8	−19.3	17.7	3	15,160	73.8	95.2	5	14,406	−1.0	−18.7	16.6	3	15,160	80.9	97.8	5	14,406
PAWPER tape	LMIC	0.8	−11.8	13.4	2	753	88.6	98.6	2	753	0.6	−12.0	13.3	2	753	88.7	98.7	2	753
Mercy method	All	−1.7	−19.5	16.2	8	5443	70.9	95.3	9	5642	−1.0	−19.7	17.7	8	5443	73.7	96.1	9	5642
Mercy method	HIC	−3.1	−20.9	14.8	5	4296	69.4	96.5	6	4495	−1.5	−20.2	17.1	5	4296	73.8	97.0	6	4495
Mercy method	LMIC	0.7	−17.2	18.7	3	1147	73.8	94.1	3	1147	0.9	−17.3	19.2	3	1147	73.2	93.6	3	1147
Wozniak method	All	−3.8	−36.1	28.5	2	628	72.1		2		−3.8	−36.3	28.6	2	628	74.9		2	1405
Wozniak method	HIC				0				0					0				0	
Wozniak method	LMIC	−3.8	−36.1	28.5	2	628	72.1		2		−3.8	−36.3	28.6	2	628	74.9		2	1405
Broselow tape	All	−1.5	−29.1	26.2	36	625,559	55.6	81.2	53	779,711	2.1	−20.6	24.9	36	625,559	61.7	91.2	53	779,711
Broselow tape	HIC	−3.9	−32.0	24.2	25	164,714	56.7	83.4	41	316,853	−4.7	−27.9	18.6	25	164,714	60.2	91.4	41	316,853
Broselow tape	LMIC	4.4	−25.9	34.7	11	462,079	52.0	76.3	12	462,530	4.6	−16.0	25.2	11	462,079	62.7	78.5	12	462,530

Data for the whole pooled sample as well as pooled data for high-income country (HIC) and low- and middle-income country (LMIC) populations are shown separately. There were very few substantial differences between the fixed effects and random effects analyses, which were not substantial enough to affect the overall outcomes. The number of studies in each pooled sample, as well as the number of data points is shown. A positive mean percentage error indicates an overestimation of weight, while a negative value indicates an underestimation of weight. Abbreviations: *MPE* mean percentage error, *LLOA* lower limit of agreement, *ULOA* upper limit of agreement, *PW10* percentage of weight estimates within 10% of actual weight, *PW20* percentage of weight estimates within 20% of actual weight

**Fig. 3:**
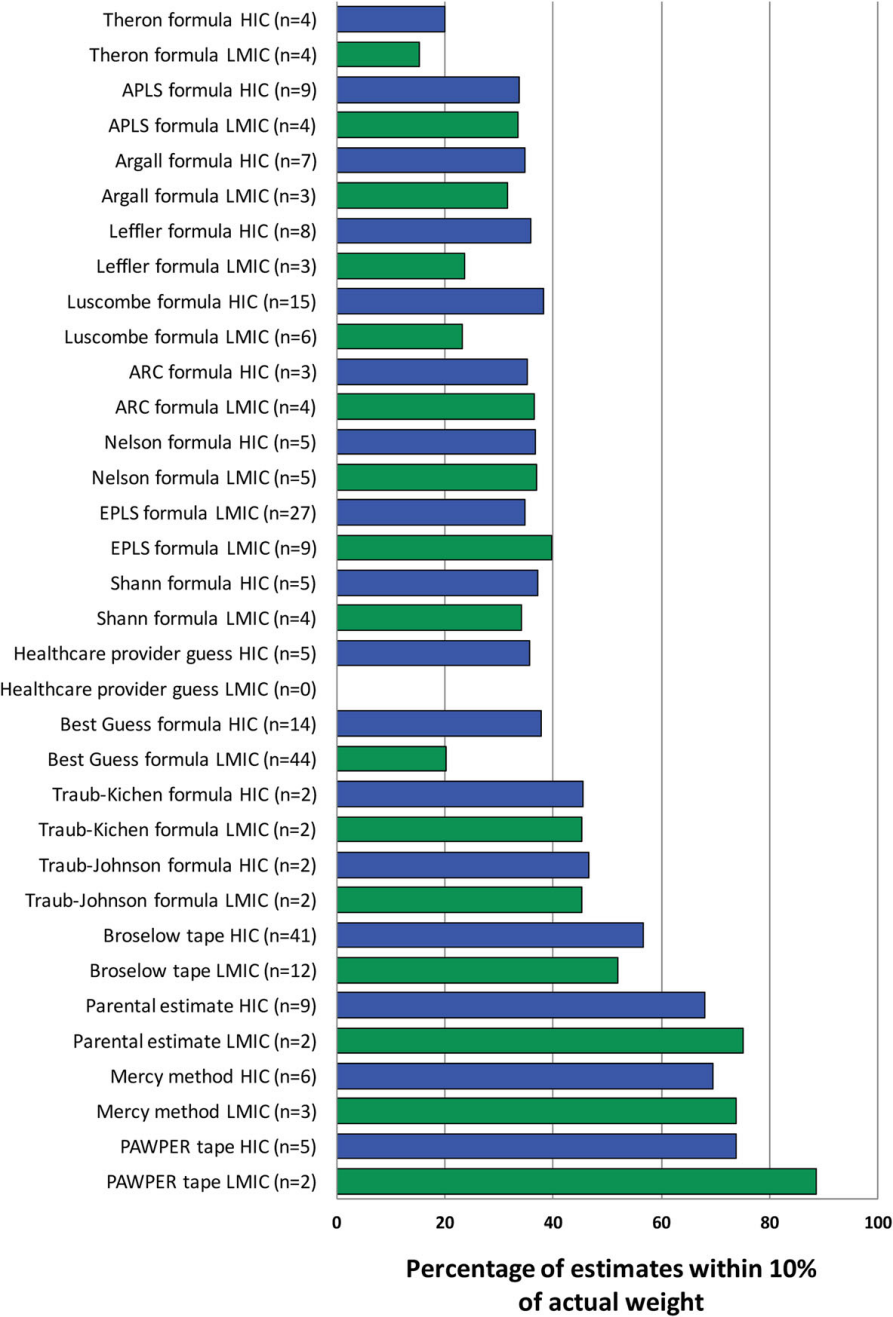
A bar chart showing the accuracy data of the major weight estimation systems evaluated

Figure 3 show the overall accuracy data for each weight estimation system (PW10 data). Age-based systems were least accurate, length-based systems were slightly more accurate and parental estimates and the two-dimensional systems were the most accurate. Despite the difference in bias between high-income countries and low- and middle-income countries for the one-dimensional systems, the overall accuracy was similarly poor. If a PW10 of 70% were used as a benchmark of acceptable accuracy, only the PAWPER tape and the Mercy method would have achieved acceptable accuracy, with parental estimates close behind. When examining the PW20 data in Table 3, only the PAWPER tape (96.6%) and the Mercy method (95.3%) met the acceptability criteria suggested by Stewart of a PW20 > 95% [30]. The PW20s for the Broselow tape, parental estimates and a value calculated for pooled age-based formulas were 81.2, 87.1 and 65.0%, respectively.

The results of the subgroup analyses are shown in Table 4.

**Table 4:** Subgroup data for each weight estimation system

	System	Subgroup		Mean percentage error	Lower limit of agreement	Upper limit of agreement	Number of children (number of studies)	PW10	Number of children (number of studies)
	APLS formula (new)	Age < 1	FE RE	19.4 8.2	−30.7 −54.5	69.4 70.8	5388 (2)	23.5 30.2	5700 (4)
		Age 1–5	FE RE	−6.0 −8.2	−45.2 −53.6	33.3 37.2	4438 (3)	37.3 35.0	8941 (5)
		Age 6–12	FE RE	7.3 3.8	−50.7 −56.2	65.2 63.8	2462 (3)	23.3 29.9	26,338 (5)
	ARC formula	Age 1–5	FE RE	−10.1 −9.8	−39.4 −39.0	19.2 19.4	1415 (2)	43.4 44.2	1415 (2)
		Age 6–10	FE RE	−12.4 −12.5	−53.4 −53.5	28.5 28.6	1405 (2)	33.2 33.3	1405 (2)
	Argall formula	Age 1–5	FE RE	3.8	−30.1	37.7	609 (1)	34.1 30.2	884 (2)
		Age 6–10	FE RE	6.8	−36.9	50.5	639 (1)	27.8 25.0	741 (2)
	Best Guess formula	Age < 1	FE RE	2.4 4.1	−42.8 −43.9	47.6 52.1	20,846 (5)	33.7 38.4	21,083 (7)
		Age 1–5	FE RE	5.0 5.1	−24.0 −20.0	34.1 30.2	46,271 (7)	48.8 44.1	50,774 (9)
		Age 6–14	FE RE	6.7 9.9	−32.5 −37.8	45.9 57.5	67,028 (6)	32.2 31.4	99,475 (9)
	Chinese age-weight rule 1	Age 1–5	FE RE	−12.2 −9.6	−51.3 −45.8	26.8 26.6	1865 (2)	33.0 35.5	1865 (2)
		Age 6–10	FE RE	−2.6 −1.5	−50.1 −47.5	44.9 44.5	1636 (2)	32.5 32.1	1636 (2)
	Chinese age-weight rule 2	Age 1–5	FE RE	−1.9 −0.9	−33.9 −32.7	30.0 30.9	1865 (2)	49.1 48.0	1865 (2)
		Age 6–10	FE RE	−4.9 −3.8	−52.1 −49.5	42.3 41.9	1636 (2)	33.9 33.3	1636 (2)
	EPLS formula	Age 1–5	FE RE	−12.7 −9.9	−43.4 −42.6	18.0 22.9	91,652 (11)	37.9 41.5	96,077 (12)
		Age 6–10	FE RE	−18.3 −19.4	−61.7 −63.8	25.0 24.9	101,742 (11)	29.0 28.1	116,988 (12)
	Leffler formula	Age < 1	FE RE	−5.7 8.8	−46.1 −42.8	34.7 60.4	20,325 (2)	36.6 27.7	20,325 (2)
		Age 1–5	FE RE	4.2 5.8	−23.4 −25.4	31.8 37.0	41,603 (3)	50.3 43.8	41,603 (3)
		Age 6–10	FE RE	−5.0 −11.0	−39.1 −45.6	29.1 23.6	64,426 (3)	42.0 31.9	64,426 (3)
	Luscombe formula	Age 1–5	FE RE	−3.3 2.4	−36.7 −32.5	30.1 37.3	47,602 (4)	43.0 37.7	52,482 (7)
		Age 6–10	FE RE	1.2 8.5	−45.3 −35.7	47.8 52.6	34,663 (4)	28.3 28.9	67,435 (7)
	Nelson formula	Age < 1	FE RE	−8.1 −3.5	−33.2 −29.2	16.9 22.2	20,217 (2)	47.9 51.1	20,217 (2)
		Age 1–5	FE RE	−9.1 −5.7	−32.9 −32.1	14.7 20.7	42,960 (3)	47.2 51.8	42,960 (3)
		Age 6–10	FE RE	−10.6 −9.4	−41.9 −46.5	20.7 27.7	64,268 (2)	38.7 37.2	64,268 (2)
	Park formula	Age < 1		2.3	−42.8	47.4	19,854 (1)	33.5	19,854 (1)
		Age 1–5		−2.7	−28.0	22.6	40,612 (1)	55.2	40,612 (1)
		Age 6–10		2.7	−34.7	40.1	63,629 (1)	39.6	63,629 (1)
	Shann formula	Age 1–5	FE RE	−2.7 −2.1	−28.1 −31.0	22.7 26.8	41,221 (2)	55.1 53.0	41,221 (2)
		Age 6–10	FE RE	−12.4 −11.8	−46.2 −50.5	21.4 26.9	64,268 (2)	34.8 35.0	64,268 (2)
	Theron formula	Age 1–5		8.2	−27.1	43.5	609 (1)	36.8	609 (1)
		Age 6–10		30.7	−12.6	74.0	639 (1)	13.5	639 (1)
Dual length- and habitus based methods	Devised weight estimating method	<10 kg	FE RE	4.0 3.7	−21.9 −21.5	29.9 28.9	221 (2)	52.7 52.5	221 (2)
		10-25 kg	FE RE	1.2 1.3	−17.7 −17.8	20.1 20.3	232 (2)	70.2 69.6	232 (2)
		>25 kg	FE RE	−4.9 −4.4	−31.9 −30.5	22.2 21.7	332 (2)	54.0 53.5	431 (2)
	PAWPER tape	<10 kg	FE RE	3.3 2.7	−13.8 −10.7	20.4 16.1	765 (4)	75.6 71.6	783 (5)
		10-25 kg	FE RE	−0.2 0.5	−13.7 −11.2	13.2 12.2	1022 (4)	86.2 85.5	1010 (4)
		>25 kg	FE RE	−2.4 −0.5	−21.7 −17.0	16.9 16.0	996 (4)	77.1 76.8	1070 (5)
	Mercy Method	<10 kg	FE RE	−1.3 −3.8	−25.1 −25.3	22.6 17.7	103 (2)	62.9 60.0	121 (3)
		10-25 kg	FE RE	−3.9 −4.4	−20.4 −20.0	12.6 11.2	218 (2)	74.3 73.0	296 (3)
		>25 kg	FE RE	−4.9 −4.3	−19.7 −18.2	9.9 9.6	311 (2)	71.1 73.4	414 (3)
	Wozniak method	<10 kg	FE RE	−21.7 −23.7	−63.7 −62.4	20.3 15.0	103 (2)	29.1 23.5	103 (2)
		10-25 kg	FE RE	0.8 0.8	−31.9 −31.6	33.5 33.1	218 (2)	69.5 68.0	218 (2)
		>25 kg	FE RE	−1.3 −0.7	−19.8 −17.6	17.2 16.2	311 (2)	78.8 81.3	311 (2)
	Broselow tape	<10 kg	FE RE	−4.4 −1.2	−29.3 −19.7	20.5 17.4	26,327 (13)	55.2 53.0	27,345 (19)
		10-25 kg	FE RE	−3.8 −1.3	−22.8 −18.9	15.1 16.3	51,915 (15)	66.4 60.7	57,102 (19)
		>25 kg	FE RE	−5.3 −2.8	−29.2 −23.9	18.6 18.2	72,803 (14)	59.7 49.9	97,639 (22)
Other	MAC formula	Age 1–5	FE RE	23.1 21.3	−8.3 −9.0	54.4 51.5	1618 (2)	17.2 20.1	1618 (2)
		Age 6–10	FE RE	0.1 0.0	−39.8 −37.3	40.0 37.4	1882 (2)	55.5 55.4	1882 (2)

The outcome data for the pooled data (not separated into high-income and low- and middle-income populations) is shown with both random effects (RE) and fixed effects (FE) results

**Fig. 4:**
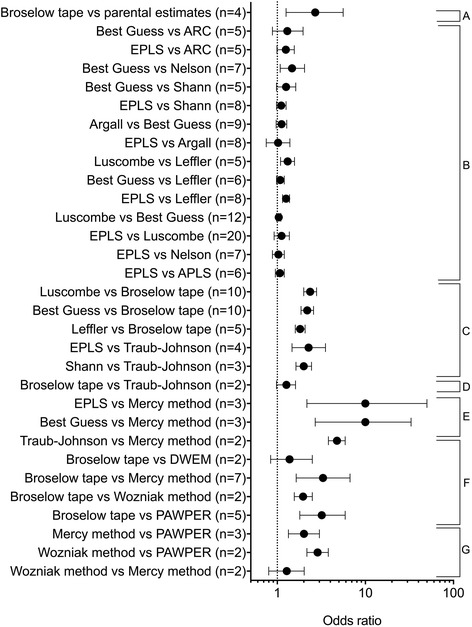
Direct meta-analysis comparisons between weight estimation systems

Figure 4 shows the results of direct statistical comparisons between weight estimation systems from studies where paired data could be pooled, using non-parametric measures of accuracy (PW10 data). The full analyses are available in Additional file 2: Figure S1. There was little difference between the accuracy of the different age formulas. Length-based methods were always more accurate than age-based methods, and two-dimensional methods were more accurate than one-dimensional methods. On direct comparison, but with data from only two studies, the PAWPER tape was significantly more accurate than the Mercy method. Parental estimates were significantly more accurate than the Broselow tape, but there was no data for direct comparison with any two-dimensional system.

## Discussion

### Summary

The quality of the evidence from the contributing studies was generally good, and the number of studies that could be included allowed for a comprehensive analysis of the data. The underlying risks of bias, while present, were considered not sufficient to alter the overall findings. Additional information on parental estimations of weight in different populations and circumstances is also required, as well as a comparison with the two-dimensional weight estimation systems.

The implications of the results for clinical practice and future research are profound: age-based formulas, along with healthcare provider guesses, were the least accurate of all weight estimation systems. They should not be used or taught. Similarly, one-dimensional length-based systems, while widely used and advocated by advanced life support organisations, were simply not accurate enough. The future challenges will be to develop two-dimensional systems, which produced the most accurate weight estimations, to be safe, quick and easy-to-use during emergency care.

Many articles on weight estimation have been—and continue to be—published without any clear indication if the results achieved, and the weight estimation systems tested, were actually good or bad. This meta-analysis has provided some useful findings which could guide researchers and decision-makers on which systems to use in clinical practice and which to explore in further research. It has also provided some perspective on the performance of weight estimation systems in high-income and low-and middle-income populations, which is important as most weight estimation systems have been developed in high-income countries and have the potential to be dangerous if used inappropriately.

### A benchmark for weight estimation systems

What degree of under- or overestimation of weight is dangerous to a child when calculating drug doses is not known [34, 35]. Many of the drugs used in paediatric emergencies have not been adequately studied to determine optimal dosing ranges. Moreover, the consequences of overestimating or underestimating weight (and therefore dose) will differ between different drugs, different patients and different clinical scenarios [36–39]. The final dose will be strongly influenced by the clinical situation and the discretion of the treating doctor, but an accurate and reliable weight estimation would still be required to provide the starting point to allow for dose modifications. Some authors regard the need for a highly accurate weight estimation as debatable. Other argue that any factors potentially impacting on patient safety must be addressed and minimised, especially in the light of compounded errors in drug dose calculations [40].

In the qualitative arm of the systematic review, we found no objective evidence to support any particular target or system by which to assess the adequacy of weight estimation methodologies. The failure to define outcome measures on how accurately a weight estimation method must perform is methodologically unsound, however. This is important as the use of a system known to be inaccurate, or inferior to another system is not good medical practice [1]. There are clearly factors other than accuracy to consider when selecting the most appropriate weight system to adopt including the complexity and cognitive load generated by the system, the vulnerability to human factor errors and its ability to interface with a drug dosing guide [41]. This needs further research.

Despite the lack of objective evidence, some reference standard is still required. A large number of articles implied or stated explicitly that an individual estimation of weight within 10% of actual weight is desirable, but only three articles provided a benchmark by which to judge a weight estimation system. The suggested criteria were that, to be considered accurate, 70% of weight estimates must be within 10% of actual weight and 95% of weight estimates must fall within 20% of actual weight [11, 30, 31]. Since the newest two-dimensional systems have shown the capability to repeatedly achieve this standard, it could, therefore, be considered a reasonable benchmark to propose to assess the adequacy of weight estimation systems in the future.

## Meta-analysis data: the accuracy of weight estimation systems

### Age-based weight estimation

The age-based formulas were the least accurate and worst-performers of all the weight estimation methods. There are multiple reasons for the inaccuracy of age-based formulas: a large biological variability in weight-for-age; a non-linear relationship between weight and age; and differences between populations with different ethnic groups and different levels of nutrition [10]. We found that age-based formulas have never been shown to perform better than length-based systems. Despite this, many authors still regard the EPLS formula as the “gold standard” for weight estimation and age-formulas are still taught on advanced life support courses [42, 43]. Some authors also still support the use of age-based formulas because of their ostensible simplicity, because they require no equipment to function and they allow advanced preparation if emergency services personnel communicate a child’s age during transport to hospital [35]. However, their use presupposes that a child’s correct age is known, that the formula is remembered correctly and that the arithmetic is performed accurately. Memory is capricious in emergencies, however, and increased stress causes errors even in calculating simple formulas [44]. The benefits of the formulas are unlikely to mitigate for their very poor accuracy [11].

Many studies have shown age-based formulas to underestimate weight in first-world populations [45–47], but studies in low- and middle-income countries have shown a significant, potentially dangerous overestimation of weight by the same formulas [48–50]. In this meta-analysis, this was confirmed, with no age-based formula performing well in any population, but the overestimation of weight in low- and middle-income populations was significant and potentially unsafe. Even the use of habitus-modified age-formulas has failed to produce an improvement in accuracy to the degree of accuracy seen with length-based habitus-modified systems, as this modification still does not account for variations in length-for-age [11, 51].

This futility of age-based weight estimation can be perfectly summed up: “Accurate paediatric weight estimation by age: mission impossible” [27]. The unavoidable conclusion is that age-based formulas should no longer be used and clinicians that manage children should ensure that a better weight-estimation system is available for use during emergency care [11, 47, 52].

### Length-based weight estimation

Every length-based system performed better than every age-based system in this study. This supports the argument that length-based weight estimation is more biologically valid than age-based estimation [10]. No length-based system achieved the acceptable outcome benchmark, however.

The two length-based formulas were originally designed to predict ideal body weight in children, but they have been used, albeit incorrectly, to estimate total body weight. The addition of a habitus-modification to these formulas has been shown to increase their performance significantly, to the same level of accuracy as the other two-dimensional systems [11]. The use of these formulas in this way shows potential, especially if used with a mobile phone app, and requires further investigation.

Although there are at least seven length-only weight-estimation tapes, only the Broselow tape has been extensively studied, while the Blantyre tape, the Sandell tape and the Handtevy tape have been evaluated only in single, small studies [53–55]. The Broselow tape, like other one-dimensional length-based systems, is vulnerable to error based on individual variations of weight-for-length (differences in body habitus) [56–59]. Some authors have questioned whether the tape is still valid given the increase in prevalence of overweight and obese children and may result in the “under-resuscitation of children” [33]. Although the manufacturer recommends modifying weight estimation up a colour zone in overweight children, to reduce this underestimation of weight, this has never been formally studied and still needs to be verified [60, 61]. However, while studies in high-income countries have demonstrated an overall underestimation of weight, studies in low- and middle-income countries have mostly shown an overestimation of weight, potentially to a dangerous degree in some populations (if drug doses were to be computed from those weights) [56, 57, 62]. Since length-based weight estimation is advocated by major, international advanced life support organisations and, since these systems are insufficiently accurate, this recommendation needs to be reconsidered and researched further [43, 63].

## Two-dimensional (dual length- and habitus-based) weight estimation

The two-dimensional systems were far superior in accuracy to the one-dimensional age- and length-based systems. The accuracies of the Mercy method and the PAWPER tape in the meta-analysis were excellent, each with a PW10 of above 70% in both over- and undernourished populations. This finding was confirmed in individual studies, with no study reporting a one-dimensional system to be more accurate than a two-dimensional method. The direct meta-analysis comparisons showed that the PAWPER and Mercy methods were significantly more accurate than the other systems, with the PAWPER tape outperforming the Mercy method in the two studies in which they were both evaluated.

All weight estimation systems have limitations, however. The Mercy method, like all other weight estimation systems was vulnerable to human factor errors in undertrained users [64]. It also has shown considerable variation in accuracy between individual assessors [19]. The functioning of the Mercy system in emergencies still needs to be evaluated– this is of concern as one of the poorest performances of the Mercy method was in a study which measured children in the supine position, as it might be used in an emergency [4, 20]. The PAWPER system was shown to be very accurate in two South African studies, one Australian study and one study based on NHANES data from the USA [5, 20–22, 31]. It was somewhat less accurate in two American studies with very obese populations, mostly because of difficulties in assessing body habitus, however [13, 65]. Although the tape’s length-based measurements are objective and simple to perform, assessment of body habitus is more subjective and dependent on training and experience [66]. This will need to be researched further to explore more standardised and objective ways of assessing habitus.

The Devised Weight Estimating Method (DWEM), the Yamamoto obesity icon system, the Wozniak system and habitus-modified Traub-Johnson and Traub-Kichen formulas have all been shown to be significantly more accurate than length-based methods, but have not yet been sufficiently studied [11, 12, 14, 67].

## Estimates of weight by parents

The utility of parental estimates of their child’s weight is dependent on the parent being willing to offer a weight estimate and being accessible to healthcare personnel at the time of the child’s need for emergency care [26]. The accuracy of prediction is determined by whether the accompanying parent is the regular caregiver of the child and whether or not the child has had a recent measurement of weight by the parent or in the parent’s presence [9]. A previous systematic review has suggested that parental estimates are the most accurate method for obtaining a weight, when it cannot be measured [9]. In this meta-analysis, parental estimates were statistically superior to the Broselow tape on direct comparison, but there were no paired data from which direct comparisons could be made with the two-dimensional systems. Only one previous study has compared the Mercy method with parental estimates, in which parental estimates were found to be more accurate [68]. This will require further research to clarify, especially the accuracy of parental estimates in populations of different socio-economic status and the frequency of availability of parental estimates. Since parents might not always be available, especially in the prehospital environment, it would be prudent to always have an alternative method of estimation available.

## Differences in weight estimation accuracy between different populations

This study showed a clear disparity in how the one-dimensional weight estimation systems performed in different populations. These differences were primarily as a result of differences in *bias*, however, while the underlying lack of *precision* within each population was similar. Thus, the variability between populations was similar to the within-population variability shown in even the most homogeneous populations. The significance of this is that, although recalibration of a system for a specific population might reduce the bias, the underlying variability and imprecision would not allow an acceptable degree of overall accuracy to be achieved. This was well shown in the study by Asskaryar et al. which failed to recalibrate the Broselow tape in an Indian population by manipulating the bias only [57]. The two-dimensional systems, with their enhanced methodology which accounts for habitus, have proven to be the closer to a universally applicable system by achieving a more uniform accuracy, both within and between populations.

## Limitations

The limitations of this study are similar to what is expected from any meta-analysis of this nature [24]. The lack of data comparing parental estimates and the newer two-dimensional systems limited the comparisons between these systems. The under-reporting on subgroups of weight status also limited the ability to analyse the performance of weight estimation systems in children with habitus that deviated from the average—this would provide insight into how the systems might function in populations with a high prevalence of underweight or obese children (or both).

## Conclusions

No evidence exists of an acceptable benchmark for weight estimation systems. An accuracy of at least PW10 > 70% and PW20 > 95% could be considered as a reference standard, since the length-based, habitus-modified systems have proven that this target is achievable across a wide range of populations.

The only weight-estimation systems that were found to be of acceptable accuracy were the two-dimensional length- and habitus-based systems. The PAWPER tape and the Mercy Method achieved an accuracy that surpassed all other methods. Wide discrepancies in the accuracy of the Broselow tape in different age groups and different populations raise questions about its use. It may dangerously overestimate weight in children from low- and middle-income countries or poor communities. Without exception, the age-based formulas evaluated proved to be highly inaccurate, with a possibility for patient harm, especially in low- and middle-income countries. There is sufficient evidence to conclude that the use of age-based formulas should be discouraged.

## Recommendations

Dual length- and habitus-based (two-dimensional) systems should be used for weight estimation in children because of superior accuracy to other systems (high quality evidence).

The Broselow tape or parental estimates of weight should be used for weight estimation in preference to age-based formulas and healthcare provider guesses (medium quality evidence).

Age-based formulas and healthcare provider guesses should not be used for weight estimation in children because of potential patient harm (high quality evidence).

Parental estimates should be used to estimate weight in preference to length-based and age-based systems (high quality evidence). There was insufficient evidence to provide a recommendation between the two-dimensional systems and parental estimates of weight.

## Additional file

Additional file 1: Table S1. Data from each study included in the meta-analysis, including subgroup data where available. (DOCX 145 kb)
Additional file 2:
**Figure S1.** (JPEG 3645 kb)

## Abbreviations

APLS: Advanced Paediatric Life Support formula
ARC: Australian Resuscitation Council formula
BG: Best Guess formula
BMI: Body mass index (kg/m^2^)
BT: Broselow tape
CAWR: Chinese age-weight rule formula
DWEM: Devised weight-estimating method
EPLS: European Paediatric Life support formula
FE: Fixed effects
LOA: 95% limits of agreement
MAC: Mid-arm circumference
MPE: Mean percentage error
NCHS: National Centre for Health Statistics
OR: Odds ratio
PW10: Percentage of estimates within 10% of actual weight
PW20: Percentage of estimates within 20% of actual weight
RE: Random effects
RMSPE: Root mean squared percentage error (%)
TJ: Traub-Johnson formula
TK: Traub-Kichen formula
