# Supplementary material for: The accuracy of emergency weight estimation systems in children—a systematic review and meta-analysis
Source: Int J Emerg Med. 2017 Sep 21;10:29. doi: 10.1186/s12245-017-0156-5 (PMC5608658; doi:10.1186/s12245-017-0156-5)
Supplement: Additional file 1: Table S1. — Data from each study included in the meta-analysis, including subgroup data where available. (DOCX 145 kb) [file 12245_2017_156_MOESM1_ESM.docx]

| **Table S1** | | | | | | | |
| --- | --- | --- | --- | --- | --- | --- | --- |
| **Ali Formula** | | | | | | | |
| **Country** | **N** | **Subgroup** | **MPE [SD] (%)** | **PELOA (%)** | **PW10 (%)** | **PW20 (%)** | **Author** |
| Trinidad 2012 | 1723 | All | -3.1 **[16.9]** | **-36.2, 30.0** | 47.5 | **75.6** | Ali |
| **Advanced Paediatric Life Support (APLS) formula (new)** | | | | | | | |
| **Country** | **N** | **Subgroup** | **MPE [SD] (%)** | **PELOA (%)** | **PW10 (%)** | **PW20 (%)** | **Author** |
| USA 2012 | 2253  1256  997 | All  1-5yrs  6-10yrs | -6.8 **[21.2]**  -12.3 **[15.7]**  0.2 **[24.9]** | -48.4, 34.8  -43.2, 18.5  -48.5, 49.0 | 36.3  42.0  29.2 | 66.7  75.1  56.1 | Cantle |
| UK 2012 | 599  184  275  102  38 | All  <1yr  1-5yrs  6-10yrs  11-12yrs | **-6.1 [36.7]**  -3.9 [39.4]  -9.4 [34.7]  -0.4 [38.7]  -8.0 [28.9] | **-78.1, 65.9**  **-81.1, 73.3**  **-77.4, 58.6**  **-76.3, 75.5**  **-64.6, 48.6** | **21.2**  **19.9**  **21.9**  **20.4**  **26.1** | **40.9**  **38.7**  **42.2**  **39.5**  **49.5** | Seddon |
| Australia 2013 | 28508  294  4425  15246  8543 | All  <1yr  1-5yrs  6-10yrs  11-14yrs |  |  | 25.1  33.3  35.5  23.4  22.7 |  | Graves |
| New Zealand 2014 | 376 | 5-10yrs |  |  | 39.1 |  | Britnell |
| Mali 2014 | 345 | All | 23.6 [21.4] | -18.3, 65.5 | 15.0 | 28.1 | Dicko |
| UK 2014 | 9436  5204  2907  1325 | All  Infants  1-5yrs  6-12yrs | **12.2 [26.2]**  **20.2 [24.5]**  **-2.9 [19.1]**  **13.6 [30.5]** | **-39.2, 63.7**  **-27.8, 68.2**  **-40.3, 34.6**  **-46.2, 73.5** | **26.8**  **23.0**  **39.5**  **23.3** | **50.8**  **44.6**  **69.9**  **44.8** | Flannigan |
| Australia 2017 | 183  18  78  68  19 | All  <1yr  1-5yrs  6-10yrs  11-14yrs |  |  | 45.8  44.4  35.9  47.1  62.9 |  | O’Leary |
| USA 2015 | 324  65  163  96 | All  <1yr  1-5yrs  6-12yrs | 18.7  15.8  16.6  21.2 |  | 36.4 | 65.7 | Chavez |
| Nigeria 2016 | 300 | All | 3.1 [18.1] | **-32.3, 38.5** | **48.2** |  | Aliyu |
| Hong Kong 2016 | 4600 | All | 0.1 | -39.8, 40.1 | 42.9 | 74.6 | So |
| Zambia 2017 | 1289 | All | 15.4 |  | 29.6 | 54.0 | Bowen |
| Thailand 2017 | 430 | All | 8.3 [26.3] | **-43.2, 59.8** | 29.5 | 54.2 | Samerchua |
| South Africa 2017 | 1066 | All | 6.1 | -40.4, 52.7 | 40.8 | 69.8 | Wells |
| **Argall formula** | | | | | | | |
| **Country** | **N** | **Subgroup** | **MPE [SD] (%)** | **PELOA (%)** | **PW10 (%)** | **PW20 (%)** | **Author** |
| Australia 2007 | 410 | All  <10kg |  |  | 37.0  85.0 |  | Nguyen |
| Croatia 2010 | 156 | All |  |  | 46.0 |  | Lulic |
| Hong Kong 2011 | 1248  609  639  463  503  282 | All  1-6yrs  7-10yrs  <20kg  20-30kg  >30kg | 5.3 **[20.1]**  3.8 **[17.3]**  6.8 **[22.3]**  10.3 **[17.1]**  11.7 **[17.2]**  -14.1 **[17.1]** | -34.1, 44.7  -30.2, 37.8  -37.0, 50.5  -23.2, 43.7  -22.0, 45.4  -47.7, 19.5 | 34.6  40.7  28.8  36.5  29.2  41.1 | 80.6  83.6  77.8  82.9  77.7  81.9 | Cattermole |
| UK 2011 | 140314 | All | -5.0 **[21.6]** | -47.4, 37.3 | 39.0 | **63.3** | Marlow |
| USA 2012 | 1013 | All | -4.6 [20.5] | **-44.8, 35.6** | 19.8 | 34.4 | Abdel-Rahman |
| UK 2012 | 377  275  102 | All  1-5yrs  6-10yrs | **-5.8 [39.1]**  -6.6 **[39.8]**  -3.5 **[37.1]** | **-82.4, 70.9**  **-84.6, 71.4**  **-76.2, 69.2** | **20.0**  **19.6**  **21.2** | **38.7**  **38.0**  **40.9** | Seddon |
| India 2014 | 249 | All | 31.5 [31.0] | **-29.3, 92.3** | 10.0 | 23.0 | Batmanabane |
| Australia 2014 | 1739 | All | 3.4 [16.1] | **-28.2, 35.0** | 46.5 |  | Allison |
| Zambia 2017 | 952 | All | 18.1 |  | 25.7 | 50.1 | Bowen |
| South Africa 2017 | 883 | All | 9.5 | -34.9, 53.9 | 36.5 | 67.3 | Wells |
| **Australian Resuscitation Council (ARC) formula** | | | | | | | |
| **Country** | **N** | **Subgroup** | **MPE [SD] (%)** | **PELOA (%)** | **PW10 (%)** | **PW20 (%)** | **Author** |
| Australia 2007 | 1572  806  766 | All  1-5yrs  6-14yrs | **-12.2 [16.7]**  -12.1 [13.3]  -12.4 [19.7] | **-45.0, 20.5**  **-38.2, 14.0**  **-51.0, 26.2** | **35.6**  **38.9**  **32.4** | **65.3**  **71.6**  **60.0** | Thompson |
| Hong Kong 2011 | 1248  609  639  463  503  282 | All  1-6yrs  7-10yrs  <20kg  20-30kg  >30kg | -10.1 **[19.8]**  -7.5 **[16.5]**  -12.5 **[22.2]**  0.1 **[13.2]**  -7.3 **[16.1]**  -31.7 **[18.3]** | -48.9, 28.7  -39.8, 24.7  -56.1, 31.2  -25.8, 25.9  -38.9, 24.3  -67.6, 4.3 | 42.1  49.4  34.1  57.0  44.7  2.5 | 83.8  87.8  80.8  90.7  84.9  68.8 | Cattermole |
| USA 2012 | 1752 | All | -16.9 [19.0] | -54.1, 20.3 | 27.5 | 51.0 | Abdel-Rahman |
| Mali 2014 | 446 | All | 9.6 [18.0] | **-25.7, 44.8** | 40.8 | 68.7 | Dicko |
| India 2014 | 350 | All | 18.1 [27.3] | **-35.4, 71.6** | 23.0 | 41.0 | Batmanabane |
| Zambia 2017 | 1071 | All | 11.1 |  | 36.8 | 64.1 | Bowen |
| South Africa 2017 | 966 | All | -2.0 [19.8] | -40.9, 36.9 | 45.3 | 75.6 | Wells |
| **Best Guess (BG) Formula** | | | | | | | |
| **Country** | **N** | **Subgroup** | **MPE [SD] (%)** | **PELOA (%)** | **PW10 (%)** | **PW20 (%)** | **Author** |
| Australia 2007 | 410  219  191 | All  1-5yrs  5-11yrs |  |  |  | **70.4**  76.0  64.0 | Kelly |
| Australia 2007 | 410 | All |  |  | 42.1 |  | Krieser |
| Australia 2007 | 1843  271  806  766 | All  <1yr  1-5yrs  6-14yrs | **5.0 [21.2]**  4.2 [23.8]  2.6 [15.6]  7.7 [24.7] | **-36.6, 46.5**  **-42.4, 50.8**  **-28.0, 33.2**  **-40.7, 56.1** | **42.3**  43.8  53.1  30.5 | 72.4  76.1  83.4  59.5 | Thompson |
| Australia 2010 | 1426  174  520  541  191 | All  <1yr  1-4yrs  5-10yrs  11-14yrs | **3.6 [19.6]**  6.3 **[21.6]**  1.7 **[15.2]**  4.5 **[20.2]**  3.4 **[25.2]** | **-34.8, 41.9**  **-36, 50**  **-28, 32**  **-35, 45**  **-46, -54** | **38.4**  **34.3**  **48.7**  **37.1**  **30.6** | **65.1**  76.0  83.0  67.0  59.0 | Casey |
| Croatia 2010 | 209 |  |  |  | 46.0 |  | Lulic |
| Hong Kong 2011 | 1248  609  639  463  503  282 | All  1-6yrs  7-10yrs  <20kg  20-30kg  >30kg | 10.1 **[20.0]**  6.2 **[16.8]**  13.9 **[22.0]**  14.1 **[14.9]**  15.6 **[20.3]**  -6.1 **[18.4]** | -29.1, 49.4  -26.8, 39.2  -29.3, 57.0  -15.2, 43.3  -24.1, 55.3  -42.1, 29.8 | 31.9  39.4  24.7  57.0  36.9  24.7 | 83.0  84.1  82.0  90.7  83.8  82.0 | Cattermole |
| South Africa 2011 | 2832  1710  1122 | All  1-5yrs  6-10yrs | 15.4 [22.0]  12.7 [19.1]  25.0 [24.0] | -**27.7, 58.5**  **-24.7, 50.1**  **-22.0, 72.0** | 31.5  40.5  17.7 | **52.9**  **60.5**  **38.7** | Geduld |
| UK 2011 | 140314 | All | 5.6 **[22.3]** | -38.0, 49.3 | 43.7 | **61.5** | Marlow |
| USA 2012 | 1563 | All | 1.0 [21.7] | **-41.5, 43.5** | 31.2 | 54.0 | Abdel-Rahman |
| Korea 2012 | 124095  19854  40612  63629 | All  Infant  Preschool  School | 5.0 [18.7]  2.3 [23.0]  4.2 [14.0]  6.3 [19.6] | **-32.1, 41.6**  **-42.9, 47.4**  **-23.3, 31.6**  **-32.1, 44.7** | **39.4**  **33.5**  **50.5**  **37.2** | **69.8**  **61.3**  **82.9**  **66.8** | Park |
| UK 2012 | 599  184  275  102  38 | All  <1yr  1-5yrs  6-10yrs  11-12yrs | **3.6 [35.8]**  3.4 **[37.4]**  4.7 **[34.0]**  1.8 **[39.7]**  2.1 **[29.2]** | **-66.6, 73.9**  **-69.9, 76.7**  **-61.9, 71.3**  **-76.0, 79.6**  **-55.1, 59.3** | **21.9**  **21.0**  **22.9**  **19.9**  **26.7** | **42.2**  **40.6**  **44.0**  **38.5**  **50.5** | Seddon |
| Australia 2013 | 37038  219  4425  15246  17148 | All  <1yr  1-5yrs  6-10yrs  11-14yrs |  |  | 24.6  29.7  36.2  22.4  23.5 |  | Graves |
| India 2014 | 347 | All | 41.3 [33.2] | **-23.8, 106.4** | 10.0 | 24.0 | Batmanabane |
| Australia 2014 | 2102  363  1739 | All  <1yr  1-5yrs | 16.4 [15.2]  4.4 [16.6]  19.0 [13.5] | **-13.4, 46.2**  **-25.2, 27.8**  **-7.5, 45.5** | 48.9  45.3  54.1 |  | Allison |
| Nigeria 2014 | 2754 | All | 21.7 [19.3] | **-16.1, 59.5** | **22.0** |  | Omisanjo |
| Australia 2015 | 199  18  78  68  35 | All  <1yr  1-5yrs  6-10yrs  11-14yrs |  |  | 47.8  61.1  51.3  47.1  31.4 |  | O’Leary |
| Hong Kong 2016 | 4600 | All | 10.9 [20.6] | -29.4, 51.3 | 38.5 | 68.2 | So |
| Zambia 2017 | 1381 | All | 30.4 |  | 16.4 | 34.3 | Bowen |
| South Africa 2017 | 1075 | All | 16.1 | -32.0, 64.2 | 32.3 | 59.2 | Wells |
| **Chinese Age Weight Rule 1 (CAWR-1)** | | | | | | | |
| **Country** | **N** | **Subgroup** | **MPE [SD] (%)** | **PELOA (%)** | **PW10 (%)** | **PW20 (%)** | **Author** |
| Hong Kong 2011 | 1248  609  639  463  503  282 | All  1-6yrs  7-10yrs  <20kg  20-30kg  >30kg | 0.8 **[20.5]**  -2.0 **[18.0]**  3.5 **[22.2]**  4.1 **[18.4]**  7.9 **[17.9]**  -17.2 **[17.2]** | -39.3, 40.9  -37.2, 33.1  -40.1, 47.2  -31.9, 40.2  -27.1, 42.9  -51.0, 16.6 | 36.1  42.4  30.2  40.0  33.2  35.1 | 80.0  81.9  78.2  81.9  78.7  79.4 | Cattermole |
| USA 2012 | 2253  1256  997 | All  1-5yrs  6-10yrs | -12.5 **[22.3]**  -17.2 **[18.9]**  -6.5 **[24.7]** | -56.1, 31.2  -54.2, 19.7  -54.8, 41.9 | 30.9  28.5  34.0 | 58.8  57.4  60.6 | Cantle |
| Hong Kong 2016 | 4600 | All | -5.2 [20.7] | -45.8, 35.3 | 35.3 | 65.6 | So |
| Zambia 2017 | 952 | All | 10.5 |  | 31.0 | 54.4 | Bowen |
| South Africa 2017 | 963 | All | 3.9 [22.6] | -40.4, 48.2 | 39.9 | 69.6 | Wells |
| **Chinese Age Weight Rule 2 (CAWR-2)** | | | | | | | |
| **Country** | **N** | **Subgroup** | **MPE [SD] (%)** | **PELOA (%)** | **PW10 (%)** | **PW20 (%)** | **Author** |
| Hong Kong 2011 | 1248  609  639  463  503  282 | All  1-6yrs  7-10yrs  <20kg  20-30kg  >30kg | 1.6 **[19.6]**  2.1 **[16.6]**  1.2 **[22.1]**  5.8 **[14.1]**  2.9 **[17.6]**  -20.5 **[18.0]** | -36.8, 40.0  -30.5, 34.7  -42.1, 44.4  -21.8, 33.5  -31.6, 37.4  -55.7, 14.7 | 38.7  44.7  30.2  49.5  41.7  25.9 | 83.3  85.4  78.2  84.0  84.7  76.6 | Cattermole |
| USA 2012 | 2253  1256  997 | All  1-5yrs  6-10yrs | -6.0 **[20.2]**  -3.9 **[15.8]**  -8.8 **[24.5]** | -45.7, 33.6  -34.8, 27.1  -56.8, 39.2 | 44.7  51.3  36.3 | 74.6  84.2  62.6 | Cantle |
| South Africa 2017 | 883 | All | 7.9 [21.0] | -33.3, 49.1 | 40.7 | 70.8 | Wells |
| **European Paediatric Life Support (EPLS) formula (original APLS)** | | | | | | | |
| **Country** | **N** | **Subgroup** | **MPE [SD] (%)** | **PELOA (%)** | **PW10 (%)** | **PW20 (%)** | **Author** |
| UK 1999 | 50 | All |  |  | 34.0 |  | Dearlove |
| Australia 2002 | 254  132  102  21 | All  10-25kg  25-40kg  >40kg | -**13.9 [16.4]**  -4.7 **[11.7]**  -20.0 **[12.9]**  -42.0 **[9.4]** | **-46.6, 18.8**  **-27.6, 18.2**  **-45.2, 5.2**  **-60.4,-23.6** | **33.4**  **57.0**  **20.9**  **0.0** | **62.6**  **88.7**  **49.9**  **1.0** | Black |
| UK / New Zealand 2005 | 237 | All | **-21.2 [24.8]** | **-69.8, 27.4** | **22.2** | **43.2** | Luscombe |
| New Zealand 2005 | 909  226  420  160  79 | All  Maori  Pacific  European  Asian | **-7.3 [30.2]**  -5.5 [33.9]  -11.1 [26.6]  -5.9 [25.7]  5.1 [40.1] | **-66.5, 52.0**  **-71.9, 60.9**  **-63.2, 41.0**  **-56.2, 44.4**  **-73.5, 83.7** | **25.2**  **22.9**  **27.0**  **29.5**  **19.5** | **48.0**  **44.0**  **51.0**  **55.2**  **37.9** | Theron |
| Australia 2007 | 410 | All |  |  | 34.0 |  | Krieser |
| UK 2007 | 13988  9010  4978 | All  1-5yrs  6-10yrs | -18.8 **[22.9]**  -**13.9 [17.9]**  **-27.9 [27.6]** | **-63.6, 26.1**  **-48.9, 21.1**  **-82.1, 26.2** | **24.6**  **32.3**  **17.3** | **47.6**  **60.4**  **34.6** | Luscombe |
| Australia 2007 | 1572  806  766 | All  1-5yrs  6-14yrs | **-15.9 [17.3]**  -12.1 [13.3]  -19.9 [19.3] | **-49.1, 17.3**  **-38.2, 14.0**  **-57.7, 17.9** | **29.9**  **38.9**  **24.3** | **57.5**  **71.6**  **48.3** | Thompson |
| Malawi 2007 | 100 | All | 10.6 **[16.8]** | **-22.3, 43.5** | **37.6** | **67.8** | Pollock |
| England 2009 | 544 | All | **-12.0 [17.3]** | **-46.0, 22.0** | **35.2** | **64.6** | Sandell |
| Hong Kong 2010 | 1370  449  473  448 | All  1-5yrs  6-8yrs  9-11yrs | -13.4 **[20.7]**  -7.4 **[16.1]**  -10.6 **[20.1]**  -22.2 **[22.6]** | -54.0, 27.2  -39.0, 24.1  -49.9, 28.7  -66.4, 22.0 | 39.0  49.2  42.9  24.6 | 83.1  87.5  84.4  77.2 | Cattermole |
| Australia 2010 | 1061  520  541 | All  1-4yrs  5-10yrs | **-15.0 [14.6]**  -12.6 **[13.0]**  -17.4 **[15.6]** | **-43.6, 13.5**  **-38, 16**  **-48, 18** | **32.3**  **38.0**  **27.8** | **64.4**  71.0  58.0 | Casey |
| Croatia 2010 | 156 | All |  |  | 36.0 |  | Lulic |
| Hong Kong 2011 | 1248  609  639  463  503  282 | All  1-6yrs  7-10yrs  <20kg  20-30kg  >30kg | -11.7 **[20.2]**  -7.5 **[16.5]**  -15.7 **[22.4]**  0.1 **[13.2]**  -8.5 **[14.4]**  -36.9 **[15.9]** | -51.2, 27.7  -39.8, 24.7  -59.6, 28.2  -25.8, 25.9  -36.8, 19.9  -68.1, -5.7 | 41.6  49.4  34.1  57.0  49.3  2.5 | 84.2  87.8  80.8  90.7  86.9  68.8 | Cattermole |
| South Africa 2011 | 2832  1710  1122 | All  1-5yrs  6-10yrs | -3.3 [17.1]  -2.4 [16.5]  -4.6 [17.9] | **-36.8, 30.2**  **-34.7, 29.9**  **-39.7, 30.5** | 57.9  58.4  57.1 | **74.9**  **77.0**  **72.1** | Geduld |
| Australia 2011 | 410 | All |  |  | 34.0 |  | Kelly |
| UK 2011 | 64197  36273  27924 | All  1-5yrs  6-10yrs | **-22.0 [23.8]**  **-16.8 [17.4]**  **-28.7 [28.8]** | **-68.6, 24.6**  **-50.9, 17.3**  **-85.1, 27.7** | **21.8**  **28.6**  **16.9** | **42.8**  **55.6**  **33.6** | Luscombe |
| UK 2011 | 140314 | All | -13.1 **[18.0]** | -48.4, 22.1 | 35.1 | **61.6** | Marlow |
| USA 2012 | 1013 | All | -14.9 [17.2] | **-48.6, 18.8** | 17.8 | 33.2 | Abdel-Rahman |
| Trinidad 2012 | 1723 | All | -5.8 **[16.9]** | **-38.9, 27.3** | 45.6 | **73.6** | Ali |
| USA 2012 | 2253  1256  997 | All  1-5yrs  6-10yrs | -17.8 **[21.1]**  -12.3 **[15.7]**  -24.6 **[24.6]** | -59.1, 23.5  -43.2, 18.5  -72.9, 23.7 | 34.8  42.0  25.7 | 64.4  75.1  51.1 | Cantle |
| Singapore 2012 | 875 | All | -7.6 **[17.4]** | -41.7, 26.5 | 45.7 | **70.6** | Loo |
| Korea 2012 | 104241  40612  63629 | All  Preschool  School | -11.5 [14.0]  -9.5 [11.9]  -13.2 [15.4] | **-38.9, 16.0**  **-32,9, 13.6**  **-43.3, 17.0** | **39.5**  **46.6**  **35.2** | **71.6**  **80.5**  **65.5** | Park |
| UK 2012 | 377  275  102 | All  1-5yrs  6-10yrs | **-12.8 [36.1]**  -9.4 **[33.8]**  -21.8 **[40.2]** | **-83.4, 57.9**  **-75.6, 56.8**  **-100.6, 57.0** | **20.5**  **22.4**  **17.0** | **39.7**  **43.1**  **33.3** | Seddon |
| Botswana 2012 | 531 | All |  |  | 37.3 |  | Wozniak |
| Australia 2013 | 19671  4425  15246 | All  1-5yrs  6-10yrs |  |  | 29.0  35.5  27.2 |  | Graves |
| Kenya 2013 | 967 | All | 5.2 **[19.0]** | **-32.0, 42.4** | **38.8** | **69.0** | House |
| Egypt 2013 | 394 | All | -0.6 **[24.3]** | **-48.2, 47.0** | 37.9 | 70.6 | Hegazy |
| USA 2013 | 207 | All |  |  | 33.0 | 72.0 | Young |
| India 2014 | 249 | All | 13.9 [24.8] | **-34.7, 62.5** | 17.0 | 45.0 | Batmanabane |
| Australia 2014 | 1739 | All | -0.1 [12.0] | **-23.6, 23.4** | 59.5 |  | Allison |
| New Zealand 2015 | 305 | All |  |  | 46.2 |  | Britnell |
| Ireland 2015 | 3155 | All | -20.3 [17.2] | -13.4, 54.0 | **23.6** | **48.3** | Skrobo |
| Japan 2016 | 237 | All |  |  | 63.8 | 89.9 | Nosaka |
| Hong Kong 2016 | 4600 | All | -8.3 [17.0] | -41.7, 25.0 | 44.6 | 76.0 | So |
| Zambia 2017 | 1071 | All | 8.1 |  | 38.0 | 66.9 | Bowen |
| Thailand 2017 | 274 | All | -4.4 [24.4] |  | 33.9 | 65.7 | Samerchua |
| South Africa 2017 | 963 | All | -4.2 [19.2] | -41.9, 33.5 | 46.8 | 75.8 | Wells |
| **Garwood Formula** | | | | | | | |
| **Country** | **N** | **Subgroup** | **MPE [SD] (%)** | **PELOA (%)** | **PW10 (%)** | **PW20 (%)** | **Author** |
| Egypt 2013 | 394 | 1-10yrs | 14.4 **[28.4]** | -**41.3, 70.1** | 35.8 | 60.9 | Hegazy |
| Zambia 2017 | 1071 | All | 20.0 |  | 24.4 | 48.0 | Bowen |
| South Africa 2017 | 996 | All | 9.2 [23.3] | -36.5, 54.8 | 35.3 | 65.9 | Wells |
| **Leffler formula** | | | | | | | |
| **Country** | **N** | **Subgroup** | **MPE [SD] (%)** | **PELOA (%)** | **PW10 (%)** | **PW20 (%)** | **Author** |
| USA 1997 | 117 | All | -13.6 [17.5] | -47.9, 20.7 | 46.2 | **61.5** | Leffler |
| UK / New Zealand 2005 | 237 | All | **-9.1 [23.4]** | **-55.0, 36.7** | **30.8** | **57.2** | Luscombe |
| New Zealand 2005 | 909  226  420  160  79 | All  Maori  Pacific  European  Asian | **-7.3 [20.3]**  -6.4 [25.6]  -11.0 [17.1]  -2.0 [18.2]  -0.5 [18.9] | **-47.1, 32.6**  **-56.6, 43.8**  **-44.5, 22.5**  **-37.7, 33.7**  **-37.5, 36.5** | **35.6**  **29.5**  **36.7**  **41.5**  **40.3** | **64.5**  **55.1**  **66.6**  **72.5**  **71.0** | Theron |
| USA 2009 | 1011  471  382  119  39  166  650  195 | All  <10kg  10-25kg  25-40kg  >40kg  UW  NW  OW | **11.0 [30.5**]  24.0 [33.0]  9.0 [17.0]  -16.0 [14.0]  -44.0 [12.0]  35.0 [40.0]  11.0 [23.0]  -11.0 [28.0] | **-48.7, 70.7**  **-40.7, 88.7**  **-24.3, 42.3**  **-43.4, 11.4**  **-67.5, 20.5**  **-43.4, 113.0**  **-34.1, 56.1**  **-65.9, 43.9** | **24.1**  **18.4**  **39.2**  **30.2**  **0.2**  **13.6**  **30.2**  **25.9** | **46.1**  **36.1**  **69.7**  **60.7**  **2.3**  **26.9**  **56.3**  **49.2** | So |
| Hong Kong 2011 | 1248  609  639  463  503  282 | All  1-6yrs  7-10yrs  <20kg  20-30kg  >30kg | -2.1 **[21.0]**  4.2 **[16.8]**  -8.1 **[22.7]**  12.2 **[12.5]**  0.2 **[13.6]**  -29.6 **[16.0]** | -43.2, 39.0  -28.8, 37.1  -52.5, 36.4  -12.3, 36.7  -26.5, 26.9  -60.9, 1.7 | 37.6  41.7  33.6  36.9  53.7  9.9 | 83.5  85.9  81.2  84.9  89.7  70.2 | Cattermole |
| UK 2011 | 140314 | All | -1.4 **[21.4]** | -43.3, 40.5 | 44.7 | **64.9** | Marlow |
| USA 2012 | 1199 | All | -5.5 [19.6] | **-43.9, 32.9** | 24.8 | 42.9 | Abdel-Rahman |
| Korea 2012 | 124095  19854  40612  63629 | All  Infant  Preschool  School | -1.7 [17.3]  -6.4 [19.7]  4.2 [14.0]  -4.9 [17.3] | **-35.6, 32.1**  **-45.1, 32.2**  **-23.3, 31.6**  **-38.7, 28.9** | **43.5**  **37.0**  **50.5**  **42.1** | **75.0**  **66.5**  **82.9**  **73.4** | Park |
| India 2014 | 247 | All | 27.8 [28.5] | **-28.1, 83.7** | 11.0 | 23.0 | Batmanabane |
| Zambia 2017 | 1024 | All | 22.4 |  | 22.5 | 44.2 | Bowen |
| South Africa 2017 | 972 | All | 7.7 [21.9] | -35.3, 50.7 | 37.6 | 68.3 | Wells |
| **Luscombe formula** | | | | | | | |
| **Country** | **N** | **Subgroup** | **MPE [SD] (%)** | **PELOA (%)** | **PW10 (%)** | **PW20 (%)** | **Author** |
| UK 2007 | 13988  9010  4978 | All  1-5yrs  6-10yrs | -2.5 **[18.7]**  -**4.5 [17.3]**  **1.1 [21.1]** | **-38.8, 34.5**  **-38.3, 29.4**  **-40.2, 42.4** | **40.4**  **42.4**  **36.4** | **71.1**  **73.7**  **65.6** | Luscombe |
| Malawi 2007 | 100 | All | 17.0 **[17.0]** | **-16.3, 50.3** | **28.4** | **55.5** | Pollock |
| Croatia 2010 | 156 | All |  |  | 46.0 |  | Lulic |
| Hong Kong 2011 | 1248  609  639  463  503  282 | All  1-6yrs  7-10yrs  <20kg  20-30kg  >30kg | 9.6 **[19.9]**  9.3 **[16.9]**  9.9 **[22.3]**  16.0 **[16.0]**  15.3 **[16.6]**  -11.1 **[17.0]** | -29.4, 48.6  -23.9, 42.4  -33.9, 53.8  -15.3, 47.2  -17.2, 47.9  -44.5, 22.3 | 30.6  35.3  26.1  28.3  25.6  43.3 | 79.8  71.3  78.4  83.2  76.1  80.9 | Cattermole |
| South Africa 2011 | 2832  1710  1122 | All  1 to 5yrs  6 to 10yrs | 12.4 [22.6]  8.6 [20.6]  22.6 [22.8] | **-31.9, 56.7**  **-31.8, 49.0**  **-22.1, 67.3** | 34.2  44.4  18.5 | **55.6**  **62.7**  **42.9** | Geduld |
| Australia 2011 | 410 | All |  |  | 45.4 |  | Kelly |
| UK 2011 | 64197  36273  27924 | All  1-5yrs  6-10yrs | **-2.1 [20.1]**  **-3.8 [16.5]**  **0.2 [23.8]** | **-41.5, 37.4**  **-36.2, 28.6**  **-46.4, 46.8** | **37.9**  **44.5**  **32.6** | **67.8**  **76.2**  **59.9** | Luscombe |
| UK 2011 | 140314 | All | 0.9 **[21.9]** | -42.1, 43.8 | 43.1 | **63.8** | Marlow |
| USA 2012 | 1013 | All | 0.8 [20.8] | **-40.0, 41.6** | 20.2 | 35.9 | Abdel-Rahman |
| Trinidad 2012 | 1723 | All | 5.0 **[19.1]** | **-32.4, 42.4** | 42.3 | **63.8** | Ali |
| Singapore 2012 | 875 | All | 7.4 **[22.2]** | -36.2, 51.0 | 37.7 | **60.6** | Loo |
| UK 2012 | 652  377  275 | All  1-5yrs  6-10yrs | -**0.5 [32.9]**  -0.5 **[31.3]**  -0.4 **[34.9]** | **-64.9, 64.0**  **-61.8, 60.8**  **-68.8, 68.0** | **23.9**  **25.1**  **22.6** | **45.7**  **47.7**  **43.3** | Seddon |
| Botswana 2012 | 531 | All |  |  | 7.2 |  | Wozniak |
| Australia 2013 | 36819  4425  15246  17148 | All  1-5yrs  6-10yrs  11-14yrs |  |  | 25.5  33.7  23.4  24.0 |  | Graves |
| USA 2013 | 207 | All |  |  | 52.0 | 84.0 | Young |
| India 2014 | 249 | All | 38.0 [31.5] | **-23.7, 99.7** | 6.0 | 14.0 | Batmanabane |
| Australia 2014 | 1739 | All | 11.1 [16.0] | **-20.3, 42.5** | 46.8 |  | Allison |
| Australia 2015 | 181  78  68  35 | All  1-5yrs  6-10yrs  11-14yrs |  |  | 47.3  38.5  47.1  37.1 |  | O’Leary |
| Ireland 2015 | 3155 | All | 3.3 [20.1] | -36.1, 42.7 | **37.6** | **67.4** | Skrobo |
| Hong Kong 2016 | 4600 | All | 7.0 [20.7] | -33.7, 47.6 | 39.8 | 69.7 | So |
| Zambia 2017 | 952 | All | 26.5 |  | 20.0 | 38.7 | Bowen |
| South Africa 2017 | 883 | All | 15.5 [23.2] | -30.0, 60.9 | 29.1 | 59.1 | Wells |
| **Nelson formula** | | | | | | | |
| **Country** | **N** | **Subgroup** | **MPE [SD] (%)** | **PELOA (%)** | **PW10 (%)** | **PW20 (%)** | **Author** |
| Hong Kong 2011 | 1248  609  639  463  503  282 | All  1-6yrs  7-10yrs  <20kg  20-30kg  >30kg | -7.9 **[19.5]**  -7.5 **[16.5]**  -8.2 **[22.0]**  0.2 **[13.4]**  -4.4 **[17.8]**  -27.3 **[17.9]** | -46.1, 30.3  -39.8, 24.7  -51.3, 34.9  -26.0, 26.4  -39.2, 30.4  -62.3, 7.7 | 42.4  49.4  35.7  57.0  42.1  18.8 | 84.2  87.8  80.8  90.1  82.5  75.9 | Cattermole |
| USA 2012 | 1399 | All | -7.7 [19.5] | **-45.9, 30.5** | 25.9 | 49.4 | Abdel-Rahman |
| Korea 2012 | 124095  19854  40612  63629 | All  Infant  Preschool  School | -10.0 [14.2]  -8.3 [12.7]  -9.5 [11.9]  -10.6 [15.9] | **-37.8, 17.9**  **-33.2, 16.5**  **-32,9, 13.8**  **-41.8, 20.5** | **42.1**  **47.8**  **46.6**  **38.7** | **74.2**  **80.9**  **80.5**  **69.6** | Park |
| Kenya 2013 | 967 | All | 10.4 **[19.0]** | **-26.8, 47.6** | **35.0** | **63.9** | House |
| Mali 2014 | 369 | All | 19.8 [21.6] | **-22.5, 62.1** | 23.5 | 40.4 | Dicko |
| Nigeria 2014 | 2754 | All | 6.4 [15.1] | **-23.2, 35.9** | 45.5 | 77.6 | Omisanjo |
| India 2014 | 329 | All | 28.2 [31.6] | **-33.7, 90.1** | 18.0 | 31.0 | Batmanabane |
| Australia 2014 | 2102  363  1739 | All  <1yr  1-5yrs | -1.2 [13.3]  1.3 [13.5]  -0.1 [12.0] | **-27.3, 24.9**  **-25.2, 27.8**  **-23.6, 23.4** | 54.8  54.3  59.5 |  | Allison |
| Zambia 2017 | 1024 | All | 11.1 |  | 36.7 | 63.7 | Bowen |
| South Africa 2017 | 1052 | All | 4.0 [21.7] | -38.6, 46.6 | 44.2 | 73.0 | Wells |
| **Park Formula** | | | | | | | |
| **Country** | **N** | **Subgroup** | **MPE [SD] (%)** | **PELOA (%)** | **PW10 (%)** | **PW20 (%)** | **Author** |
| Korea 2012 | 124095  19854  40612  63629 | All  Infant  Preschool  School | 0.9 [18.2]  2.3 [23.0]  -2.7 [12.9]  2.7 [19.1] | **-34.9, 36.6**  **-42.9, 47.4**  **-28.0, 22.6**  **-34.7, 40.1** | **41.7**  **33.5**  **55.2**  **39.6** | **72.8**  **61.3**  **87.1**  **70.0** | Park |
| Japan 2016 | 237 | All |  |  | 45.1 | 75.5 | Nosaka |
| Zambia 2017 | 1381 | All | 25.0 |  | 21.9 | 42.9 | Bowen |
| South Africa 2017 | 1075 | All | 10.9 [23.9] | -36.0, 57.8 | 39.0 | 66.0 | Wells |
| **Shann formula** | | | | | | | |
| **Country** | **N** | **Subgroup** | **MPE [SD] (%)** | **PELOA (%)** | **PW10 (%)** | **PW20 (%)** | **Author** |
| New Zealand 2005 | 909  226  420  160  79 | All  Maori  Pacific  European  Asian | **-0.8 [33.9]**  -2.3 [37.9]  -3.7 [29.9]  1.8 [28.9]  13.9 [44.9] | **-67.2, 65.6**  **-76.6, 72.0**  **-62.3, 54.9**  **-54.8, 58.4**  **-74.1, 101.9** | **23.2**  **20.8**  **26.0**  **27.0**  **16.8** | **44.5**  **40.2**  **49.3**  **51.0**  **32.9** | Theron |
| Hong Kong 2011 | 1248  609  639  463  503  282 | All  1-6yrs  7-10yrs  <20kg  20-30kg  >30kg | -6.5 **[20.3]**  -1.5 **[16.6]**  -11.2 **[22.3]**  6.3 **[12.8]**  -3.8 **[14.3]**  -32.1 **[16.2]** | -46.3, 33.4  -34.1, 31.1  -55.0, 32.7  -18.7, 31.4  -31.8, 24.2  -63.8, -0.5 | 42.7  50.7  35.1  53.6  53.3  6.0 | 83.7  87.4  80.3  88.1  87.7  69.5 | Cattermole |
| USA 2012 | 1752 | All | -17.4 [20.9] | **-58.4, 23.7** | 26.3 | 48.6 | Abdel-Rahman |
| Korea 2012 | 104241  40612  63629 | All  Preschool  School | -8.6 [16.4]  -2.7 [12.9]  -12.4 [17.2] | **-40.7, 23.5**  **-27.9, 22.6**  **-46.1, 21.3** | **40.6**  **55.2**  **34.8** | **71.6**  **87.1**  **64.1** | Park |
| Egypt 2013 | 394 | 1-10yrs | -5.3 **[26.3]** | **-56.8, 46.2** | 39.8 | 65.7 | Hegazy |
| India 2014 | 350 | All | 18.4 [27.1] | **-34.7, 71.5** | 22.0 | 42.0 | Batmanabane |
| New Zealand 2015 | 305 | All |  |  | 53.1 |  | Britnell |
| Zambia 2017 | 1071 | All | 16.1 |  | 30.7 | 56.4 | Bowen |
| South Africa 2017 | 996 | All | 1.5 [21.0] | -39.7, 42.7 | 43.8 | 71.3 | Wells |
| **Theron formula** | | | | | | | |
| **Country** | **N** | **Subgroup** | **MPE [SD] (%)** | **PELOA (%)** | **PW10 (%)** | **PW20 (%)** | **Author** |
| USA 2009 | 1011  471  382  119  39  166  650  195 | All  <10kg  10-25kg  25-40kg  >40kg  UW  NW  OW | **56.0 [77.4]**  106.0 [85.0]  12.0 [26.0]  20.0 [34.0]  -10.0 [23.0]  101.0 [105.0]  56.0 [69.0]  15.0 [48.0] | **-95.9, 207.6**  **-60.6, 273**  **-40.0, 63.0**  **-46.6, 86.6**  **-55.1, 35.1**  **-105.0, 307.0**  **-79.2, 191.0**  **-79.1, 109.0** | **7.9**  **4.3**  **27.1**  **19.6**  **30.8**  **4.8**  **8.3**  **15.7** | **15.8**  **8.7**  **51.2**  **38.0**  **57.2**  **9.6**  **16.6**  **30.9** | So |
| Hong Kong 2011 | 1248  609  639  463  503  282 | All  1-6yrs  7-10yrs  <20kg  20-30kg  >30kg | 19.7 **[23.1]**  8.2 **[18.0]**  30.7 **[22.1]**  14.7 **[25.5]**  27.9 **[25.1]**  13.3 **[21.5]** | -25.5, 65.0  -27.0, 43.3  -12.6, 74.0  -22.1, 51.6  -21.2, 77.0  -28.9, 55.4 | 24.8  36.8  13.5  33.0  16.1  27.0 | 84.7  83.6  85.8  87.3  84.7  80.5 | Cattermole |
| USA 2012 | 1013 | All | 9.6 [26.4] | **-42.1, 61.3** | 19.6 | 33.4 | Abdel-Rahman |
| Botswana 2012 | 531 | All |  |  | 8.5 |  | Wozniak |
| India 2014 | 249 | All | 51.4 [42.6] | **-32.1, 134.9** | 7.0 | 14.0 | Batmanabane |
| New Zealand 2015 | 305 | All |  |  | 27.5 |  | Britnell |
| Zambia 2017 | 920 | All | 27.0 |  | 19.9 | 37.3 | Bowen |
| South Africa 2017 | 1085 | All | 29.2 [37.3] | -43.9, 102.4 | 25.4 | 45.4 | Wells |
| **Guesses by healthcare providers (HCPs)** | | | | | | | |
| **Country** | **N** | **Subgroup** | **MPE [SD] (%)** | **PELOA (%)** | **PW10 (%)** | **PW20 (%)** | **Author** |
| USA 1990 | 138 | All |  |  |  |  | Hughes |
| England 1997 | 75 | All | **1.3 [34.4]** | **-66.1, 68.7** | **22.9** | **43.9** | Greig |
| USA 1999 | 100 | All | -5.9 **[19.2]** | -43.5, 34.6 | **38.1** | **68.0** | Harris |
| Malawi 1999 | 142 | All |  |  |  | 54.0 | Molyneux |
| Japan 2002 | 48 | All |  |  |  | **57.5** | Uesegi |
| USA 2009 | 704 | All |  |  | 56.0 |  | Partridge |
| Australia 2010 | 468 | All |  |  | **17.1** | **45.7** | Williams |
| USA 2011 | 372 | All | -2.8 [21.8] | **-45.5, 39.9** | 44.0 | 63.7 | Rosenberg |
| USA 2013 | 179 | All |  |  |  | 82.4 | Lim |
| **Estimates by parents** | | | | | | | |
| **Country** | **N** | **Subgroup** | **MPE [SD] (%)** | **PELOA (%)** | **PW10 (%)** | **PW20 (%)** | **Author** |
| USA 1997 | 117 | All | -6.8 [9.8] | -26.0, 12.4 | 80.3 | **90.8** | Leffler |
| UK 1999 | 50 | All |  |  | 25.0 |  | Dearlove |
| Israel 1999 | 233  138 | All  < 5yrs | 0.1 [18.5] | **-36.2, 36.3** | 73.4  **41.1** | 87.5  **72.0** | Goldman |
| USA 1999 | 100 | All | -1.3 **[19.5]** | -38.9, 37.7 | **39.1** | **69.4** | Harris |
| Australia 2007 | 410 | All |  |  | 78.0 |  | Krieser |
| USA 2009 | 777 | All |  |  | 79.0 |  | Partridge |
| Belgium 2011 | 71 | All |  |  |  |  | Huybrechts |
| Thailand 2012 | 595  111  372  104  8 | All  <10kg  20-25kg  25-40kg  >40kg |  |  | 85.2  80.2  87.1  84.6  75.0 |  | Trakulsrichai |
| USA 2013 | 207 | All |  |  | 54.0 | 79.0 | Young |
| Japan 2016 | 237 | All |  |  | 94.9 | 98.7 | Nosaka |
| Thailand 2017 | 344 | All | -2.6 [7.1] | -16.5, 11.3 | 88.7 | 97.4 | Samerchua |
| **Broselow Tape** | | | | | | | |
| **Country** | **N** | **Subgroup** | **MPE [SD] (%)** | **PELOA (%)** | **PW10 (%)** | **PW20 (%)** | **Author** |
| USA 1988  BT 1998 | 937  395  449  93 | All  3.5-10kg  10-25kg  >25kg |  |  | 59.7  55.9  65.0  49.5 | 89.6  85.6  95.1  83.9 | Lubitz |
| UK 1999  *BT version NR* | 50 | All |  |  | 60.0 |  | Dearlove |
| Hong Kong 2000  *BT version NR* | 909  129  631  149 | All  <10kg  10-25kg  >25kg |  |  | 69.5  56.6  76.2  52.3 | 91.3  83.7  95.2  81.2 | Kun |
| Australia 2002  *BT version NR* | 339  121  132  86 | All  <10kg  10-25kg  25-40kg | **-2.0 [11.7]**  -0.6 **[12.3]**  -0.4 **[9.4]**  -6.4 **[12.8]** | **-24.9, 20.9**  **-24.7, 23.5**  **-18.8, 20.9**  **-31.5, 18.7** | **60.0**  **58.3**  **71.2**  **51.1** | **90.8**  **89.6**  **96.6**  **83.6** | Black |
| Switzerland 2002  *BT version NR* | 585  339  246 | All  ≤20kg  >20kg |  |  | 65.0  66.0  65.0 |  | Hofer |
| New Zealand 2005  *BT version NR* | 909  226  420  160  79 | All  Maori  Pacific  European  Asian | **-9.5 [12.6]**  -11.1 [13.6]  -11.0 [11.0]  -5.9 [12.5]  -3.9 [14.0] | **-34.1, 15.1**  **-37.9, 15.6**  **-32.6, 10.5**  **-30.4, 18.7**  **-31.2, 23.5** | **45.5**  **40.7**  **43.6**  **52.7**  **50.8** | **78.8**  **73.2**  **79.1**  **85.1**  **83.1** | Theron |
| USA 2006  BT1998 | 7813  2305  2660  2848 | All  <10kg  10-20kg  >20kg | -3.9 [11.9]  -**3.6 [12.7]**  **-5.0 [10.9]**  **-3.8 [15.6]** | -**27.2, 19.4**  **-28.4, 21.3**  **-26.4, 16.5**  **-34.4, 26.7** | 55.3  57.0  59.8  53.2 | 86.6  **87.0**  **90.5**  **78.7** | Nieman |
| USA 2006  BT2005A | 7671  2305  2660  2706 | All  <10kg  10-20kg  >20kg | -5.6 [11.9]  **-2.9 [11.9]**  **-5.7 [9.5]**  **-7.8 [13.4]** | -**28.9, 17.7**  **-26.3, 20.5**  **-24.4, 13.0**  **-34.0, 18.5** | 60.0  62.3  62.0  58.5 | 93.7  **89.7**  **93.0**  **80.0** | Nieman |
| USA 2007  *BT version NR* | 300  100  100  100 | All  <10kg  10-20kg  20-36kg | **-8.2 [19.1]**  -9.9 [15.5]  -7.1 [11.3]  -7.5 [26.9] | **-45.6, 29.3**  **-40.3, 20.5**  **-29.3, 15.0**  **-60.1, 45.1** | **36.7**  **40.3**  **53.6**  **27.9** | **66.2**  **71.6**  **86.5**  **52.6** | DuBois |
| USA 2007  BT2002B | 1207  544  520  143 | All  Birth  4-6yrs  10-12yrs |  |  |  | 66.2  100  70.0  40.6 | Hashikawa |
| Korea 2007  *BT version NR* | 665 | All  ≤26kg  >26kg |  |  | 57.9  64.5  38.5 |  | Jang |
| Australia 2007  *BT version NR* | 410 | All |  |  | 61.0 |  | Krieser |
| India 2008  *BT version NR* | 548  175  197  176 | All  <10kg  10-18kg  >18kg | **9.0 [13.1]**  2.4 [12.6]  11.3 [10.9]  12.9 [13.5] | **-16.8, 34.7**  **-22.3, 27.1**  **-10.1, 32.7**  **-13.6, 39.4** | **43.6**  52.6  44.7  33.5 | **78.6**  **88.1**  **78.6**  **69.3** | Ramarajan |
| Ireland 2009  *BT version NR* | 545 | All | -8.6 [13.2] | -34.5, 17.2 | 69.7 | 86.6 | Anstett |
| India 2009  *BT version NR* | 15000  4256  6076  4668 | All  <10kg  10-18kg  >18kg | **-11.6**  -2.4  -13.3  -17.9 |  | 63.0  57.0 |  | Cattamanchi |
| USA 2009  *BT version NR* | 1011  471  382  119  39  166  650  195 | All  <10kg  10-25kg  25-40kg  >40kg  UW  NW  OW | -**4.2 [21.6]**  0.2 [24.0]  -3.7 [16.0]  -12.0 [17.0]  -38.0 [12.0]  24.0 [29.0]  -3.0 [10.0]  -28.0 [12.0] | **-46.4, 38.1**  **-46.8, 47.2**  **-35.1, 27.7**  **-45.3, 21.3**  **-61.5, -14.5**  **-32.8, 80.8**  **-22.6, 16.6**  **-51.5, 43.9** | **35.0**  **32.3**  **45.7**  **35.5**  **1.0**  **19.4**  **66.1**  **6.6** | **63.6**  **59.5**  **77.7**  **65.1**  **6.7**  **38.1**  **94.5**  **25.2** | So |
| Australia 2009  *BT version NR* | 475  71  316  88 | All  <10kg  10-25kg  >25kg | -5.5 [12.9] | -31.2, 20.4 | 62.0  68.0  63.0  53.0 | 90.0  88.0  93.0  81.0 | Stewart |
| Australia 2010  *BT version NR* | 1235  174  520  541 | All  <1yr  1-4yrs  5-10yrs | **-6.3 [14.6]**  -7.2 [15.0]  -7.0 **[11.7]**  -5.3 **[16.7**] | **-34.8, 22.3**  **-36, 22**  **-30, 16**  **-38, 58** | **46.8**  **44.8**  **52.8**  **43.1** | **88.1**  83.0  91.0  87.0 | Casey |
| Hong Kong 2010  BT1998 | 1199  449  471  279 | All  1-5yrs  6-8yrs  9-11yrs | -1.9 **[14.1]**  -1.3 **[13.2]**  -1.9 **[14.2]**  -2.9 **[15.3]** | -29.5, 25.7  -27.2, 24.7  -29.7, 25.9  -32.8, 27.1 | 58.4  65.3  56.7  56.3 | 89.0  91.5  90.1  83.0 | Cattermole |
| Croatia 2010  *BT version NR* | 151 | All |  |  | 58.0 |  | Lulic |
| Canada 2011  *BT version NR* | 243 | All | -11.9 **[14.9]** | -41.1, 17.3 | 48.1 | 81.5 | Bourdeau |
| South Africa 2011  *BT version NR* | 2832  1710  1122 | All  1-5yrs  6-10yrs | -0.9 [14.3]  -2.4 [13.8]  1.6 [14.7] | **-28.9, 27.1**  **-29.4, 24.6**  **-27.2, 30.4** | 64.2  63.5  65.4 | **83.7**  **84.7**  **82.4** | Geduld |
| USA 2011  BT2007B | 657 | All |  |  |  | 46.9 | Knight |
| USA 2011  BT2007B | 372 | All | -3.0 [15.3] | **-33.0, 27.0** | 63.0 | **80.0** | Rosenberg |
| USA 2012  *BT version NR* | 1214 | All | -6.1 [12.3] | **-30.1, 18.0** | 37.4 | 53.9 | Abdel-Rahman |
| Singapore 2012  BT2007B | 875 | All | -0.6 **[14.0]** | -28.1, 26.8 | 58.9 | **72.3** | Loo |
| Canada 2012  BT2002A | 6361 | All | -7.1 [16.9] | -40.2, 26.0 | 56.3 | 84.5 | Milne |
| Korea 2012  *BT version NR* | 124095  19854  40612  63629 | All  Infant  Preschool  School | -4.8 [10.9]  -4.9 [12.2]  -4.0 [9.0]  -5.5 [11.7] | **-26.1, 16.5**  **-28.8, 19.0**  **-21.6, 13.6**  **-28.4, 17.4** | **59.6**  **55.1**  **68.8**  **55.7** | **90.7**  **87.1**  **95.8**  **87.8** | Park |
| USA 2012  *BT version NR* | 118 | All | -8.4 **[8.3]** | **-24.7, 7.9** | 50.0 | **91.9** | Sinha |
| Thailand 2012  *BT version NR* | 595  111  372  104  8 | All  <10kg  10-25kg  25-40kg  >40kg |  |  | 56.1  60.4  59.7  43.3  0.0 |  | Trakulsrichai |
| Botswana 2012  *BT version NR* | 758 | All |  |  |  | 55.0 | Wozniak |
| USA 2013  *BT version NR* | 415 | All | -4.1 [11.9] | **-27.4, 19.2** | 58.6 | 90.8 | Abdel-Rahman |
| Iran 2013  *BT version NR* | 403 | All |  |  | 72.5 |  | Akabarian |
| Sudan 2016  *BT version NR* | 583  227  141  215 | All  Well nourish  Malnourish  Sev malnour |  |  |  | **15.1**  28.2  8.5  6.7 | Clark |
| Australia 2013  BT2007B | 24426  294  4421  14028  5683 | All  <1yr  1-5yrs  6-10yrs  11-14yrs |  |  | 49.4  47.3  55.7  49.7  43.8 |  | Graves |
| Kenya 2013  BT2007B | 967 | BT 2007B | 2.2 **[12.7]** | **-22.7, 27.1** | **56.2** | **87.9** | House |
| Korea 2013 | 105072  104998  102316 | BT 2005 A  BT 2007 B  BT 2011 A | -4.5  -3.9  -0.5 |  | 64.8  65.6  67.7 |  | Suh |
| South Africa 2013  BT2007B | 453  120  193  140 | All  <12kg  12-20kg  >20kg | -3.8 [11.2]  -2.4 [10.5]  -3.0 [10.2]  -6.0 [12.6] | **-25.8, 18.2**  **-23.0, 18.2**  **-23.0, 17.0**  **-30.7, 18.7** | 63.6  65.8  65.8  58.6 | 91.6  93.3  93.8  87.1 | Wells |
| USA 2013  *BT version NR* | 207 | All |  |  | 56.0 | 81.0 | Young |
| Mali 2014  *BT version NR* | 365 | All | 8.2 [10.4] | **-12.2, 28.6** | 41.2 | 67.4 | Dicko |
| India 2014  *BT version NR* | 321 | All | 10.8 [16.3] | **-21.1, 42.7** | 28.0 | 60.0 | Batmanabane |
| Thailand 2014  BT2007A | 3869 | All | -3.6 [12.9] | **-28.9, 12.9** | 58.0 |  | Chiengkriwate |
| Australia 2014  BT2007B | 2102  363  1739 | All  <1yr  1-5yrs | -0.4 [11.0]  -5.9 [10.8]  0.6 [9.3] | **-25.7, 24.9**  **-27.5, 15.7**  **-20.6, 21.8** | **63.6**  **57.7**  **71.7** |  | Allison |
| Mexico 2015  *BT version NR* | 815  236  343  236 | All  <10kg  10-18kg  >18kg | -0.9 [15.9]  0.5 [20.8]  -2.6 [12.1]  -0.0 [15.1] | **32.1, 30.3**  **-40.3, 41.3**  **-26.3, 21.1**  **-29.6, 29.6** | 55.3  46.2  64.1  51.5 |  | Khouli |
| New Zealand 2015  BT2011A | 305 | All |  |  | 73.4 |  | Britnell |
| India 2015  BT2007B | 769 | All | 8.5 [14.6] | **-20.1, 37.1** | **43.8** |  | Asskaryar |
| India 2015 | 416 | All | **0.3 [13.8]** | **-26.6, 27.3** | **53.1** |  | Asskaryar |
| South Africa 2016  BT2011A | 300  73  128  99 | All  <12kg  12-20kg  >20kg | 5.5 [13.4]  7.3 [12.5]  6.3 [11.2]  3.3 [16.3] | **-20.8, 31.8**  **-17.2, 31.8**  **-15.7, 28.3**  **-28.6, 35.2** | 54.0  60.3  53.1  50.5 | 84.3  86.3  87.5  78.8 | Georgoulas |
| Australia 2017  *BT version NR* | 160  18  78  60  5 | All  <1yr  1-5yrs  6-10yrs  11-14yrs |  |  | 68.2  50.0  73.1  54.4  91.4 |  | O’Leary |
| South Africa 2017  BT2011A | 328  30  90  212 | All  <12kg  12-20kg  >20kg | -5.1 [16.2]  4.8 [8.9]  -0.7 [12.3]  -8.4 [17.4] | -36.9, 26.6  -12.7, 22.3  -24.8, 23.5  -42.5, 25.6 | 49.4  73.3  60.0  45.8 | 79.9  93.3  91.1  73.6 | Wells |
| USA 2015  *BT version NR* | 324  65  163  96 | All  <1yr  1-5yrs  6-12yrs | 13.0  18.7  10.0  14.8 |  | 52.5 | 81.4 | Chavez |
| Nigeria 2016  *BT version NR* | 300 | All | 3.1 [19.5] | **-35.2, 41.3** | **46.3** |  | Aliyu |
| Korea 2016  BT2011A | 906 | All | 6.4 **[18.0]** | **-31.1, 39.5** | 60.6 |  | Jung |
| USA 2016  BT2011A | 2434 | All |  |  | 54.4 |  | Lowe |
| Japan 2016  BT2007B | 237 | All |  |  | 75.9 | 97.5 | Nosaka |
| Multinational 2016  BT2007B  BT2011A | 453990 | All | 4.6 [10.4] | **-15.8, 25.0** | 62.9 | 95.6* | Ralston |
| Malaysia 2016  *BT version NR* | 1163 | All | 2.6 [20.7] | **-37.9, 43.2** | **36.8** |  | Sahar |
| Zambia 2016  *BT version NR* | 1282 | All | 4.3 |  | 47.3 | 75.1 | Bowen |
| Thailand 2017 | 341 | All | 4.5 [15.4] | **-25.7, 34.7** | 46.3 | 78.0 | Samerchua |
|  |  |  |  |  |  |  |  |
| **Devised Weight Estimating Method (DWEM)** | | | | | | | |
| **Country** | **N** | **Subgroup** | **MPE [SD] (%)** | **PELOA (%)** | **PW10 (%)** | **PW20 (%)** | **Author** |
| USA 1986 | 258 | All  <20kg |  |  | 61.0  66.0 |  | Garland |
| Australia 2002 | 484  121  132  121  110 | All  <10kg  10-25kg  25-40kg  >40kg | **0.6 [11.5]**  7.0 **[11.2]**  0.7 **[8.8]**  -2.0 **[12.3]**  -3.9 **[10.7]** | **-22.0, 23.1**  **-15.0, 29.0**  **-16.5, 17.9**  **-26.1, 22.1**  **-24.9, 17.1** | **61.5**  **54.1**  **74.3**  **57.8**  **61.9** | **91.8**  **86.9**  **97.7**  **89.1**  **92.1** | Black |
| USA 2007 | 400  100  100  100  100 | All  <10kg  10-20kg  20-36kg  >36kg | **-2.8 [14.2]**  0.4 [14.5]  1.8 [10.6]  -6.7 [12.8]  -6.6 [16.0] | **-30.5, 25.0**  **-28.0, 28.8**  **-18.9, 22.5**  **-31.8, 18.3**  **-37.9, 24.8** | **51.0**  **50.9**  **64.8**  **50.6**  **43.4** | **83.3**  **83.2**  **93.7**  **83.2**  **75.1** | DuBois |
| **PAWPER Tape** | | | | | | | |
| **Country** | **N** | **Subgroup** | **MPE [SD] (%)** | **PELOA (%)** | **PW10 (%)** | **PW20 (%)** | **Author** |
| South Africa 2013 | 453  120  193  140 | All  <12kg  12-20kg  >20kg | 0.0 [6.2]  0.9 [6.1]  0.0 [5.9]  -0.9 [6.8] | **-12.2, 12.2**  **-11.1, 12.9**  **-11.6, 11.6**  **-14.2, 12.4** | 89.2  90.8  90.2  86.4 | 99.1  100  99.0  98.7 | Wells |
| South Africa 2016 | 300  73  128  99 | All  <12kg  12-20kg  >20kg | 1.6 [6.7]  4.5 [7.6]  1.0 [6.0]  0.4 [6.2] | **-11.5, 14.7**  **-10.4, 19.4**  **-10.8, 12.8**  **-11.8, 12.6** | 87.7  78.1  93.0  87.9 | 98.3  95.9  99.2  99.0 | Georgoulas |
| Australia 2015 | 170  18  78  64  10 | All  <1yr  1-5yrs  6-10yrs  11-14yrs |  |  | 77.1  55.6  80.8  72.1  88.6 |  | O’Leary |
| South Africa 2017 | 328  30  90  212 | All  <12kg  12-20kg  >20kg | 1.1 [7.7]  6.4 [6.5]  1.2 [5.7]  0.3 [8.2] | -13.9, 16.1  -6.2, 19.1  -10.1, 12.4  -15.8, 26.5 | 83.4  70.0  90.0  82.5 | 98.5  100  100  97.6 | Wells |
| USA 2015 | 324  65  163  96 | All  <1yr  1-5yrs  6-12yrs | 10.1  13.1  8.2  11.5 |  | 63.0 | 89.2 | Chavez |
| USA 2015 | 1698  542  611  545 | All  <12kg  12-20kg  >20kg | -2.6 **[12.1]**  4.2 **[11.1]**  -2.4 **[8.6]**  -9.5 **[12.5]** | **-26,3, 21.1**  **-17.6, 26.0**  **-19.3, 14.5**  **-34.0, 15.0** | 63.5  63.8  77.9  47.0 |  | Garcia |
| USA 2017 | 13134 | All | -0.9 [8.5] | **-17.5, 15.7]** | 81.9 | 98.0 | Wells |
| **Mid-arm circumference (MAC) formula** | | | | | | | |
| **Country** | **N** | **Subgroup** | **MPE [SD] (%)** | **PELOA (%)** | **PW10 (%)** | **PW20 (%)** | **Author** |
| Hong Kong 2010 | 1370  449  473  448 | All  1-5yrs  6-8yrs  9-11yrs | 2.9 **[17.5]**  17.2 **[15.2]**  -0.5 **[14.1]**  -7.9 **[12.7]** | -31.4, 37.2  -12.5, 47.0  -28.0, 27.1  -32.9, 17.0 | 44.2  26.5  53.9  51.8 | 85.1  79.7  88.6  86.8 | Cattermole |
| USA 2012 | 2130  1169  961 | All  1-5yrs  6-10yrs | 15.7 **[21.1]**  25.3 **[15.7]**  4.1 **[24.6]** | -18.4, 49.8  -5.1, 55.7  -18.4, 26.6 | 33.5  13.6  57.8 | 56.7  31.3  87.6 | Cantle |
| Botswana 2012 | 777 | All |  |  | 32.2 |  | Wozniak |
| USA 2012 | 529 | All | 3.5 [12.1] | **-20.2, 27.2** | 15.5 | 23.9 | Abdel-Rahman |
| USA 2015 | 324  65  163  96 | All  <1yr  1-5yrs  6-12yrs | 28.8  >100  33.2  12.4 |  | 24.1 | 40.1 | Chavez |
| Multinational 2016  BT2007B  BT2011A | 453990 | All | 4.6 [10.4] | **-15.8, 25.0** | 27.9 | 61.8* | Ralston |
| **Mercy Method** | | | | | | | |
| **Country** | **N** | **Subgroup** | **MPE [SD] (%)** | **PELOA (%)** | **PW10 (%)** | **PW20 (%)** | **Author** |
| USA 2012 | 1938 | All | -0.5 [8.4] | **-16.8, 16.0** | 78.6 | 98.0 | Abdel-Rahman |
| USA 2013 | 624 | All | 1.7 [8.5] | **-15.0, 18.4** | 76.4 | 98.1 | Abdel-Rahman |
| USA 2013 | 976 | All | -0.3 [9.4] | -18.7, 18.1 | 77.0 | 95.4 | Abdel-Rahman |
| Mali 2014 | 473 | All | 1.6 [9.3] | **-16.6, 19.8** | 71.5 | 96.7 | Dicko |
| India 2014 | 374 | All | 1.5 [9.9] | **-17.9, 20.9** | 70.0 | 96.0 | Batmanabane |
| South Africa 2016 | 300  73  128  99 | All  <12kg  12-20kg  >20kg | -0.9 [8.3]  2.3 [10.6]  -1.5 [7.7]  -2.6 [6.4] | **-17.2, 15.4**  **-18.5, 23.1**  **-16.6, 13.6**  **-15.1, 9.9** | 80.1  69.9  82.0  84.8 | 98.2  91.8  100  100 | Georgoulas |
| Australia 2015 | 199  18  78  68  35 | All  <1yr  1-5yrs  6-10yrs  11-14yrs |  |  | 69.7  66.7  69.2  72.1  65.7 |  | O’Leary |
| Thailand 2017 | 430 | All | -9.6 [10.9] |  | 50.7 | 82.3 | Samerchua |
| South Africa 2017 | 328  30  90  212 | All  <12kg  12-20kg  >20kg | -6.7 [8.3]  -9.9 [11.3]  -7.3 [8.2]  -6.0 [7.8] | -23.1, 9.6  -32.1, 12.3  -23.3, 8.7  -21.4, 9.4 | 63.9  43.3  67.8  65.1 | 94.3  86.7  91.1  96.7 | Wells |
| **Wozniak method** | | | | | | | |
| **Country** | **N** | **Subgroup** | **MPE [SD] (%)** | **PELOA (%)** | **PW10 (%)** | **PW20 (%)** | **Author** |
| Botswana 2012 | 777 | MAC-ulna  MAC-tibia |  |  | 80.8  81.1 |  | Wozniak |
| South Africa 2016 | 300  73  128  99 | All  <12kg  12-20kg  >20kg | -3.6 [15.7]  -18.9 [22.4]  1.0 [9.8]  1.0 [7.0] | -**34.4, 27.2**  **-62.8, 25.0**  **-18.2, 20.2**  **-12.7, 14.7** | 70.6  37.0  76.6  87.9 | 88.3  65.8  93.8  98.0 | Georgoulas |
| South Africa 2017 | 328  30  90  212 | All  <12kg  12-20kg  >20kg | -4.0 [17.3]  -28.5 [17.1]  0.5 [23.2]  -2.4 [10.2] | -37.8, 29.9  -62.0, 5.1  -45.1, 46.0  -22.3, 17.5 | 64.5  10.0  58.9  74.5 | 84.0  26.7  88.9  90.1 | Wells |
| **Oakley System** | | | | | | | |
| **Country** | **N** | **Subgroup** | **MPE [SD] (%)** | **PELOA (%)** | **PW10 (%)** | **PW20 (%)** | **Author** |
| Australia 2002 | 442  121  132  121  68 | All  <10kg  10-25kg  25-40kg  >40kg | **5.4 [16.5**]  16.0 **[14.0]**  10.0 **[9.4]**  -1.0 **[14.6]**  -11.0 [**16.8]** | **-26.9, 37.7**  **-11.4, 43.4**  **-8.4, 28.4**  **-29.6, 27.6**  **-43.9, 21.9** | **43.4**  **30.2**  **48.3**  **50.6**  **37.1** | **75.0**  **60.7**  **85.6**  **82.8**  **67.1** | Black |
| New Zealand 2005 | 909  226  420  160  79 | All  Maori  Pacific  European  Asian | **-16.2 [16.9]**  -15.8 [20.1]  -19.8 [14.5]  -10.3 [15.1]  -10.8 [17.0] | **-49.2, 16.8**  **-55.1, 23.6**  **-48.1, 8.6**  **-40.0, 19.3**  **-44.0, 22.5** | **29.6**  **28.7**  **23.0**  **40.3**  **37.1** | **57.3**  **54.5**  **50.2**  **71.7**  **67.1** | Theron |
| **Growth Chart methods** | | | | | | | |
| **Country** | **N** | **Subgroup** | **MPE [SD] (%)** | **PELOA (%)** | **PW10 (%)** | **PW20 (%)** | **Author** |
| England 2009 | 544 | All | -4.0 **[12.5]** | -29.0, 20.0 | **55.3** | **87.2** | Sandell |
| Thailand 2012 | 595 | All |  |  | 51.4 |  | Trakulsrichai |
| **Sandell tape** | | | | | | | |
| **Country** | **N** | **Subgroup** | **MPE [SD] (%)** | **PELOA (%)** | **PW10 (%)** | **PW20 (%)** | **Author** |
| Australia 2014 | 2102  363  1739 | All  <1yr  1-5yrs | 12.4 [11.7]  13.3 [13.7]  12.2 [11.0] | **-10.5, 35.3**  **-13.6, 40.2**  **-9.4, 33.8** | 60.7  53.5  63.7 |  | Allison |
| **Hanging-leg weight technique** | | | | | | | |
| **Country** | **N** | **Subgroup** | **MPE [SD] (%)** | **PELOA (%)** | **PW10 (%)** | **PW20 (%)** | **Author** |
| USA 1990 | 100 | All  <10kg  10-25kg  >25kg | 9.0 |  | 73.7  50.0  80.0  90.0 |  | Haftel |
| **Finger Counting system** | | | | | | | |
| **Country** | **N** | **Subgroup** | **MPE [SD] (%)** | **PELOA (%)** | **PW10 (%)** | **PW20 (%)** | **Author** |
| USA 2013 | 207 | All |  |  | 59.0 | 87.0 | Young |
| Hong Kong 2016 | 4178 | All | 0.1 [14.8] | -34.0, 34.2 | 48.6 | 79.7 | So |
| **Traub-Johnson (TJ) formula** | | | | | | | |
| **Country** | **N** | **Subgroup** | **MPE [SD] (%)** | **PELOA (%)** | **PW10 (%)** | **PW20 (%)** | **Author** |
| Australia 2002 | 374  132  121  121 | All  10-25kg  25-40kg  >40kg | **-7.1 [13.6]**  -2.6 **[9.4]**  -4.0 **[13.5]**  -15.0 **[14.0]** | **-33.6, 19.5**  **-21.0, 15.8**  **-30.5, 22.5**  **-42.4, 12.4** | **48.0**  **69.4**  **52.2**  **32.3** | **80.5**  **96.0**  **84.4**  **63.3** | Black |
| USA 2012 | 1752 | All | -7.1 [15.8] | **-38.1, 23.9** | 45.2 | 69.3 | Abdel-Rahman |
| India 2014 | 350 | All | 11.1 [16.0] | **-20.3, 42.5** | 29.0 | 62.0 | Batmanabane |
| South Africa 2017 | 1085 | All | -0.2 [13.2] | -26.2, 25.7 | 61.3 | 87.3 | Wells |
| **Traub-Kichen (TK) formula** | | | | | | | |
| **Country** | **N** | **Subgroup** | **MPE [SD] (%)** | **PELOA (%)** | **PW10 (%)** | **PW20 (%)** | **Author** |
| Australia 2002 | 374  132  121  121 | All  10-25kg  25-40kg  >40kg | -**7.5 [14.4]**  -0.7 **[8.8]**  -6.5 **[12.3]**  -16.0 **[16.8]** | **-35.8, 20.7**  **-17.9, 16.5**  **-30.6, 17.6**  **-48.9, 16.9** | **45.7**  **74.3**  **52.2**  **30.0** | **77.9**  **97.7**  **84.8**  **57.8** | Black |
| USA 2012 | 1747 | All | -9.5 [15.9] | **-40.7, 21.7** | 45.3 | 67.8 | Abdel-Rahman |
| India 2014 | 344 | All | 10.0 [16.4] | **-22.1, 42.1** | 28.0 | 63.0 | Batmanabane |
| South Africa 2017 | 990 | All | -0.8 [12.6] | -25.4, 23.9 | 62.5 | 88.8 | Wells |

Data from each study included in the meta-analysis, including subgroup data where available. Imputed data is indicated by bold type.
