# Supplementary figures and images for: The accuracy of emergency weight estimation systems in children—a systematic review and meta-analysis
Source: Int J Emerg Med. 2017 Sep 21;10:29. doi: 10.1186/s12245-017-0156-5 (PMC5608658; doi:10.1186/s12245-017-0156-5)

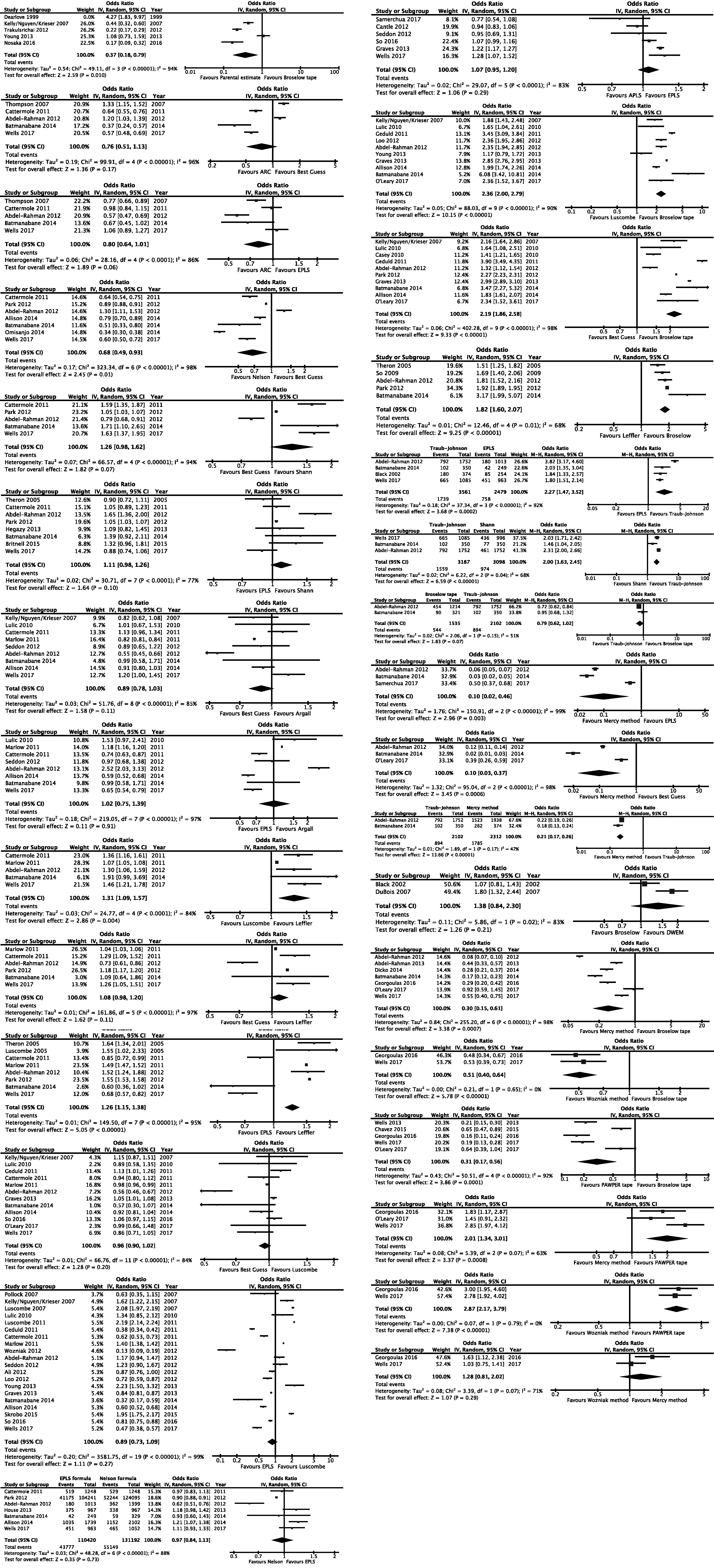

Supplement: Additional file 2: — Figure S1. (JPEG 3645 kb) [file 12245_2017_156_MOESM2_ESM.jpeg]
